# Dynamics of Droplets Impacting on Aerogel, Liquid Infused, and Liquid-Like Solid Surfaces

**DOI:** 10.1021/acsami.2c14483

**Published:** 2022-12-29

**Authors:** Jack Dawson, Samual Coaster, Rui Han, Johannes Gausden, Hongzhong Liu, Glen McHale, Jinju Chen

**Affiliations:** † School of Engineering, Newcastle University, Newcastle Upon TyneNE1 7RU, United Kingdom; ‡ School of Mechanical Engineering, Xi’an Jiaotong University, Xi’an710054, China; § School of Engineering, Institute for Multiscale Thermofluids, The University of Edinburgh, EdinburghEH9 3FB, United Kingdom

**Keywords:** droplet impact, SLIPS, SOCAL, aerogel, superhydrophobic, friction, adhesion

## Abstract

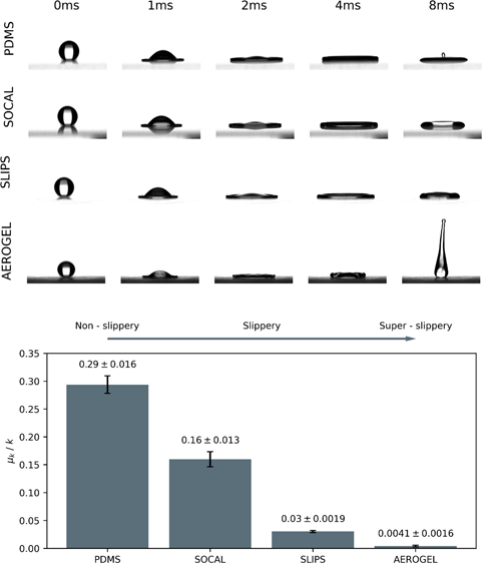

Droplets impacting superhydrophobic surfaces have been extensively studied due to their compelling scientific insights and important industrial applications. In these cases, the commonly reported impact regime was that of complete rebound. This impact regime strongly depends on the nature of the superhydrophobic surface. Here, we report the dynamics of droplets impacting three hydrophobic slippery surfaces, which have fundamental differences in normal liquid adhesion and lateral static and kinetic liquid friction. For an air cushion-like (super)hydrophobic solid surface (Aerogel) with low adhesion and low static and low kinetic friction, complete rebound can start at a very low Weber (*We*) number (∼1). For slippery liquid-infused porous (SLIP) surfaces with high adhesion and low static and low kinetic friction, complete rebound only occurs at a much higher *We* number (>5). For a slippery omniphobic covalently attached liquid-like (SOCAL) solid surface, with high adhesion and low static friction similar to SLIPS but higher kinetic friction, complete rebound was not observed, even for a *We* as high as 200. Furthermore, the droplet ejection volume after impacting the Aerogel surface is 100% across the whole range of *We* numbers tested compared to other surfaces. In contrast, droplet ejection for SLIPs was only observed consistently when the *We* was above 5–10. For SOCAL, 100% (or near 100%) ejection volume was not observed even at the highest *We* number tested here (∼200). This suggests that droplets impacting our (super)hydrophobic Aerogel and SLIPS lose less kinetic energy. These insights into the differences between normal adhesion and lateral friction properties can be used to inform the selection of surface properties to achieve the most desirable droplet impact characteristics to fulfill a wide range of applications, such as deicing, inkjet printing, and microelectronics.

## Introduction

Superhydrophobic surfaces have a wide range of scientific and industrial applications. They can be created by fabricating micro- or nanopatterned structures on low surface energy materials.^1,2^ Such surfaces retain air in their structure to form air pockets that minimize direct solid–liquid contact.^3−5^ Aerogel—a porous structure containing over 99% air^6,7^—is one such example. Hydrophobic aerogels have been proven as promising materials in various applications such as oil/water separation^8^ and absorption of organic matter like oil.^9−11^ Under high water pressure, the liquid may repel the air, leading to the decay of the hydrophobic characteristics.

Another category of hydrophobic slippery surface has been developed by replacing the air trapped in structured surfaces with a low surface tension nonvolatile and immiscible lubricating liquid. In slippery liquid infused porous surfaces (SLIPS)^12^—a specific type of liquid-infused surface (LIS)^13^—the infused liquid is trapped in the pores of the surface structure by interfacial forces and provides a continuous layer of liquid acting as a lubricant at the surface. This leads to a smooth and homogeneous liquid surface with a small contact angle hysteresis. SLIPS exhibit self-cleaning,^2,14−18^ self-healing,^19−21^ anti-icing properties,^19,22−24^ and antibiofouling performance.^15,18,25−27^ However, the potential loss of lubricant through repeated usage or shear^28−30^ remains a key limiting factor to broader adoption as a practical solution. Therefore, another hydrophobic slippery surface, known as a slippery omniphobic covalently attached liquid-like (SOCAL) solid surface, has been proposed.^31,32^ SOCAL is obtained through acid-catalyzed graft poly condensation of dimethyl-dimethoxysilane and was first proposed by Wang and McCarthy as an ultraslippery nonpinning surface for sessile droplets.^31,32^ The SOCAL surface displays similar static wetting properties to SLIPS through its grafted polydimethylsiloxane (PDMS) coating that behaves as a liquid phase approximately 150 °C above its glass transition temperature.^31,33^ SOCAL does not suffer from shear-induced depletion of the lubricant and has demonstrated more sustainable antibiofilm performance in constant flow than SLIPS.^34^

Recently it has been suggested that, although SLIPS and SOCAL both have similar static contact angles and low contact angle hysteresis, droplets on SOCAL exhibit low mobility and high dynamic (sometimes referred to as kinetic) friction.^35,36^ This difference in the dynamic properties of droplets on these two surfaces reflects the recent observation that the friction properties, sometimes called the “lateral adhesion”, of droplets on surfaces can be divided into a static and a kinetic regime similar to the static and kinetic friction regimes for solids sliding on solid surfaces.^37^ For solids sliding on solids, these concepts are summarized in Amontons’ laws, which state that the friction force is proportional to the normal load force with the constant of proportionality given by either a coefficient of static friction or a coefficient of kinetic friction.^38,39^ For a droplet on a surface, there is an Amontons’-like law *F*_f_ = *μF*_N_ relating the frictional force *F*_f_, to the normal force due to the vertical component of the surface tension force, i.e., *F*_N_ = *πwγ*_LV_ sin *θ*_e_, where *w* is the droplet diameter, *γ*_LV_ is the (droplet) liquid–vapor surface tension, and *θ*_e_ is the equilibrium contact angle.^40^ In this formulation of droplet friction, the coefficient of static friction, *μ*_s_, is directly proportional to the contact angle hysteresis, and the coefficient of kinetic friction, *μ*_k_, is directly proportional to the difference in contact angles at the front and back of the droplet when it is in motion. Since the reaction of a surface to the normal component of the surface tension force is adhesive, the Amontons’-like law for droplets implies a direct relationship between liquid adhesion in a direction normal to the surface and the friction (or resistance) to motion along the surface. Amontons’-like laws for droplets on surfaces and coefficients of friction were developed by considering the advancing and receding motion of contact lines.

From the above discussion on the relationship between the normal adhesion and the friction felt by droplets on surfaces, we hypothesize that a relationship may also exist between the kinetic friction and the adhesion felt by droplets impacting and rebounding from surfaces. In particular, relationships may exist depending on whether surfaces have lower or higher adhesion and whether these surfaces display lower or higher kinetic friction against droplet motion. In this work, we regard hydrophobic aerogel as a low adhesion and low static and low kinetic friction surface due to its superhydrophobic contact angle, typically above 150°, and the high mobility of droplets sliding on and impacting against its surface. The hydrophobic aerogel is distinguished from both SLIP and SOCAL surfaces, which have high normal adhesion due to their contact angles, typically around 100° (as evidenced by their ability to support hanging droplets). We also expect SOCAL surfaces to be distinguished from SLIPS due to their higher kinetic friction, which can alter the energy available for rebound after the spreading and contraction phase of the impacting droplet process.

The understanding of the fundamental characteristics of droplet impact on these three hydrophobic, but slippery, surfaces is important at both a basic level in relation to adhesion and friction and also in determining their future applications in scenarios such as inkjet printing,^41^ spray coating,^42,43^ spray cooling,^44−46^ and anti-icing.^47^ For context, previous work has been done to investigate droplets impacting solid surfaces with different architecture and roughness, and some work has been conducted to study droplet impact on either SLIPS^48^ or SOCAL.^36^ However, none of these works have studied droplets impacting different slippery surfaces such as (super)hydrophobic silica Aerogel, SLIPS, and SOCAL and sought to understand the differences in impact behavior. This work will pave the way for understanding the surface wetting of these three fundamentally different slippery surfaces.

## Materials and Methods

### Specimen Fabrication

Samples of PDMS were produced using an elastomer kit SYLGARD 184 (Dow Corning Corporation, Midland, MI). Base and curing agent components were mixed thoroughly (10:1 wt/wt ratio), and entrapped air was removed by degassing in a vacuum chamber for 30 min. This mixture was then decanted into the wells of a custom mold (each well was an 18 × 18 × 3 mm^3^ cuboid) and cured overnight in a 60 °C oven. Once cured, samples were removed from the mold, sonicated for 20 min to remove large surface contaminants, and sterilized in an autoclave. The samples were then stored in a Petri dish until they were used.

To produce the SLIPS studied here, several sterile PDMS samples were placed in a six-well plate and submerged in silicone oil (10cSt, 0.93 g/mL, Sigma-Aldrich) overnight. Before testing, excess oil was drained from each sample by placing it on its side on the well-plate rim for 2 min. Pooled oil was removed by gentle wiping with a lens tissue. The thickness of each oil layer was calculated using a Python script, which solved eq 1 below, using measurements taken before and after swelling and after thorough wiping of the sample surface.^49^
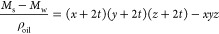
1In eq 1, *M*_s_ and *M*_w_ represent the swollen and wiped mass, respectively; *ρ*_oil_ is the density of the silicone oil; *x*, *y*, and *z* are the dimensions of the sample post swelling; and *t* is the thickness of the lubricant layer to be found. The oil reserve within the PDMS can be calculated using the preswelling mass and dimensions; however, this is not explored here.

SOCAL surfaces were created on 25 × 75 mm^2^ glass slides using the method detailed by Wang and McCarthy.^31^ The protocol employed here was further optimized by Armstrong et al.^33^ In short, glass slides were sonicated in 10% Decon 90 and DI water and then placed into a Henniker plasma cleaner (HPT-100) at 30% power for 20 min to add OH bonds to their surface. These slides were then dipped into a reactive solution of isopropanol, dimethyl-dimethoxysilane, and sulfuric acid (90, 9, and 1% wt) for 5 s and then slowly withdrawn. These slides were then placed into a bespoke humidity chamber in a controlled environment (60% relative humidity, 25 °C) for 20 min. The acid-catalyzed graft polycondensation of dimethyl-dimethoxysilane creates a homogeneous layer of PDMS chains grafted to the glass surface. The excess unreacted material was rinsed away with deionized (DI) water, isopropanol, and toluene.

The superhydrophobic silica aerogel samples (Hydrophobic Silica Disc, SKU: P-AT.SIO2.HP.100.D.1IN.) were purchased from Aerogel Technologies, LLC, Boston, MA. Measurements of sample mass and dimensions were used to calculate the volume fraction of air within each sample. The surface roughness of all the solid surfaces was measured using atomic force microscopy.

### Surface Wetting Characterization

The wetting characteristics of each of the four samples (i.e., the three types of slippery hydrophobic samples and the PDMS sample) tested were characterized by static contact angle (CA), contact angle hysteresis (CAH), and droplet kinetic friction measurement. Static CA was measured by placing a sample onto the stage of a bespoke goniometer and depositing an 8 μL droplet of deionized (DI) water onto its surface. A camera and microscope lens were used to take images of each droplet, and contact angles were extracted by droplet edge fitting in a custom Python script. Mean values were calculated from 15 measurements per surface (3 samples, 5 locations each).

**Figure 1:**
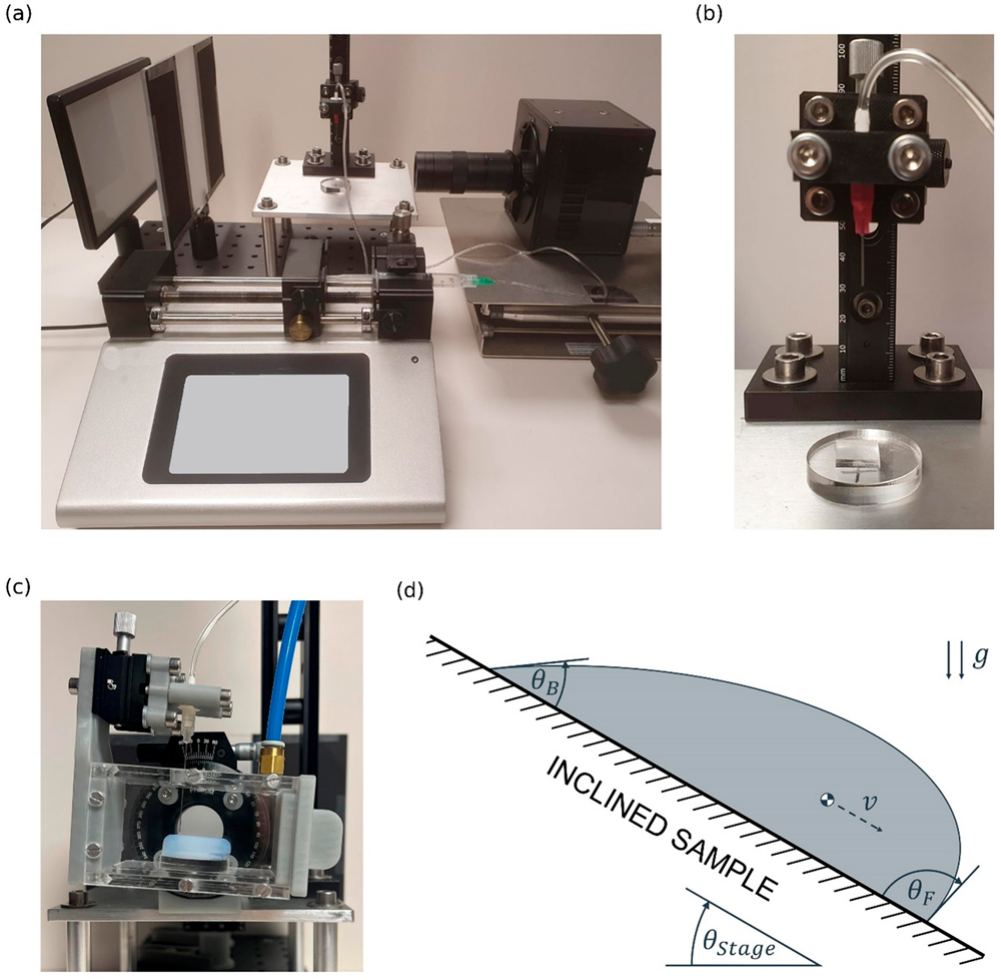
(a) Image of the custom droplet impact imaging stage. (b) Close-up of the needle holder, sample stage, and height rail. (c) Image of the tilting stage equipment used to carry out droplet kinetic friction measurements. (d) Diagram showing the front and back contact angles of a droplet sliding on an inclined surface at an arbitrary velocity.

Following static angle measurement, the CAH was measured using the protocol outlined by Barrio-Zhang et al.^36^ In short, each 8 μL droplet was inflated by 4 μL at a rate of 0.2 mL/min using a needle and syringe pump (like that shown in Figure 1b), and a series of images were captured for 2 min (5 frames/s) while the droplet relaxed. After this, the droplet was deflated by 4 μL, and another series of images were captured for 2 min. Droplet edge fitting was performed on each inflation and deflation image series to get the advancing and receding contact angles. CAH was computed as the difference between the advancing and receding angles, and a mean was calculated from 15 measurements per surface (3 samples, 5 locations each). The difference in advancing and receding contact angles indicates the resistance to initiating droplet motion on each surface (i.e., static friction).

Finally, the kinetic friction experienced by droplets sliding on each surface was measured using the tilting stage equipment shown in Figure 1c. For tests on PDMS and SOCAL, where large droplets were used, a given sample was first leveled, after which a droplet of DI water was deposited onto its surface using a 27-gauge needle. Deposition on a leveled surface was carried out to ensure a droplet could be entirely deposited without causing premature sliding due to the forced motion of its contact points. Once entirely deposited, the needle was removed, the sample stage was inclined to initiate droplet sliding, and a video sequence (50fps) was captured using a high-speed camera (Photron FASTCAM Mini UX50). For SLIPS and Aerogel, where smaller droplets could initiate fast sliding at shallow stage angles, the stage was inclined prior to droplet deposition; in these tests, removal of the needle initiated sliding. The droplet volumes and stage angles used in these tests are provided in Table 1.

**Table 1:** Droplet Volumes and Stage Angles Used in Droplet Sliding Tests

	PDMS	SOCAL	SLIPS	Aerogel
*v*_drop_ (μL)	30	25	7	7
*θ*_stage_ (deg)	40	30	10	5

For all tests, a custom python script was utilized to extract the back and forward contact angles (see *θ*_B_ and *θ*_F_ in Figure 1d) for all frames of each video, and the ratio of the coefficient of kinetic friction, *μ*_k_, to dimensionless shape factor, *k*, was calculated for each frame using eq 2 below:^40^
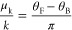
2where *θ*_B_ and *θ*_F_ are in radians. The results were taken when *θ*_F_ – *θ*_B_ almost reached equilibrium; this value has been reported to be a constant at low speeds.^37,40^ An average *μ*_k_/*k* was then calculated for each video sequence and means and standard deviations were calculated across these values for all 15 tests performed on each sample (3 samples, 5 locations each).

### Droplet Impact Testing and Analysis

A bespoke droplet impact stage was employed in all impact tests—shown in Figure 1a,b. In each test, 8 μL droplets of deionized water were released above a given surface from a 25-gauge needle fed by a 3 mL syringe and syringe pump at a rate of 0.2 mL/min. A total of 12 different drop heights were used: varying between 5 mm (*V* ≅ 0.15 ms^–1^) and 550 mm (*V* ≅ 3.2 ms^–1^). The droplet stage was illuminated by a cold white light (VILTROX, L116T LED Light), and the droplets’ falls and impacts were recorded using a Photron FASTCAM Mini UX50 at 5000 fps.

To analyze impact tests, each image series was first reviewed in ImageJ to get timing information (contact time, bounce time, etc.), droplet size and velocity information, and the pixel coordinates of the sample surface. This information was then input into a custom edge-fitting Python script—alongside each image series—to calculate important test parameters such as the droplets’ spread and bounce evolution after surface contact (defined in a later section). Due to minor differences in the initial size and velocity of impacting droplets, the dimensionless Weber and Reynolds numbers are used to compare individual tests in this study. These are defined in the literature by eqs 3 and 4, respectively, below:
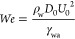
3
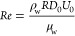
4where *We* is the Weber number; *Re* is the Reynolds number; *ρ*_w_ is the density of the water droplet (≅996 kg·m^–3^); *U*_0_ and *D*_0_ are the initial speed and diameter of the droplet as it falls toward the surface, respectively; *γ*_wa_ is the surface tension at the air–water interface (≅72 mN·m^–1^);^50^ and *μ*_*w*_ is the dynamic viscosity of water at room temperature (≅0.001 Pa·s).

### Statistical Analysis

Tabulated data are presented as mean values with standard error. One-way ANOVA was applied, and *p < 0.05, **p < 0.01, ***p < 0.001, and ****p < 0.0001 were considered statistically significant in this study. Representative curves of spreading and bounce ratios are provided instead of averages to prevent loss of meaning.

## Results and Discussion

### Surface Wettability

**Figure 2:**
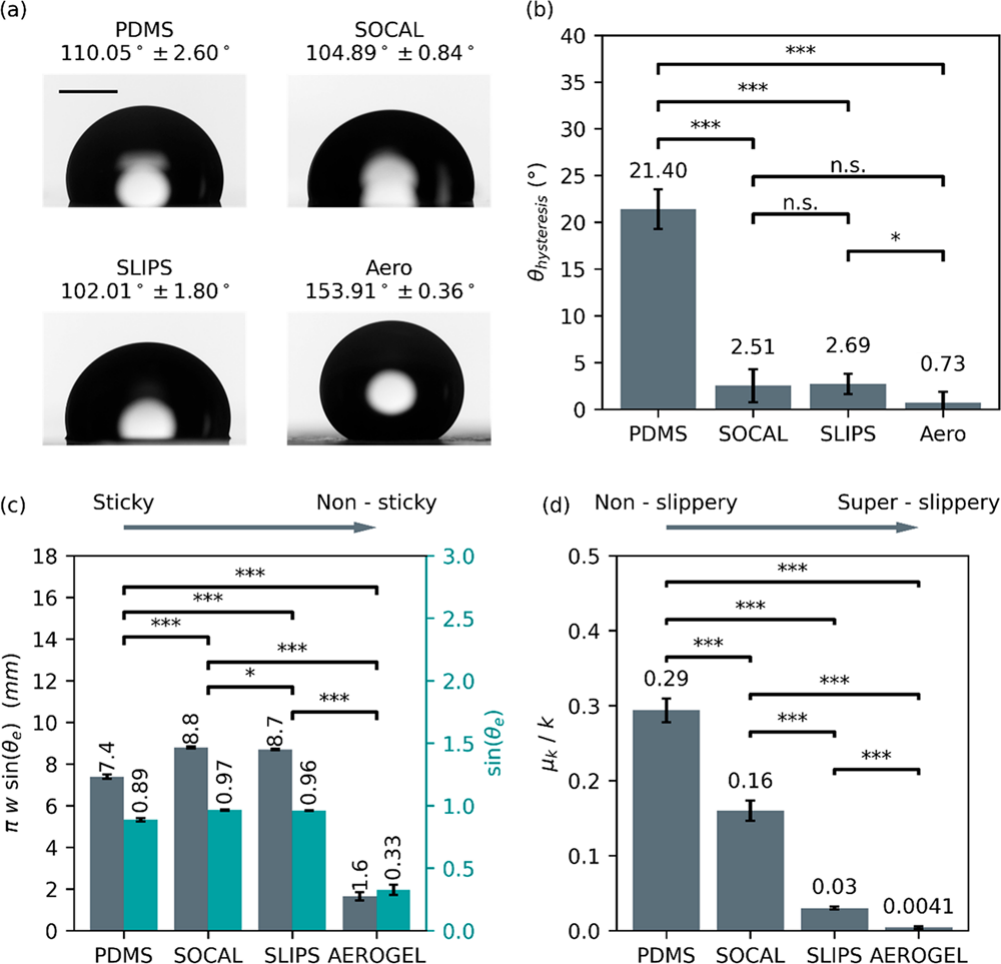
Comparison of the (a) static contact angle, *θ*_s_, and (b) contact angle hysteresis, *θ*_hysteresis_, of DI water droplets deposited on all four surfaces tested (PDMS, SLIPS, SOCAL, and Aerogel). A 1 mm scale bar is provided in the first image of subfigure (a), and each CA value is provided as a mean ± std. (c) Comparison of values of π*w* sin *θ*_e_ = *πw* (sin *θ*_F_ + sin *θ*_B_)/2 for each surface, where *θ*_F_ and *θ*_B_ are defined in Figure 1d. (d) Comparison of the droplet kinetic friction quantified using *μ*_k_/*k*, for all four surfaces. * represents a student’s *t* test *p*-value <0.05, ** < 0.001, and *** < 0.0001. The kinetic friction of Aerogel presented in (d) was calculated using angles measured from both the compressed and relaxed stages of droplet bouncing (see Video S1 of Aerogel bouncing in the Supporting Information).

Figure 2a provides selected snapshots of static droplets on each surface and a comparison of the static CA measured for each surface. All angles shown in Figure 2a are significantly different (student’s *t* test *p*-value <0.05). Figure 2b provides a comparison of the CAH of droplets of DI water deposited onto the surface of each of the samples tested in this study. The oil layer thickness of the SLIPS samples prepared in this study was 17.8 ± 1.7 μm, based on eq 1. The air volume fraction of the Aerogel samples was measured to be 0.936 ± 0.009. As shown in Figure 2a,b, the plain PDMS samples tested were more hydrophobic than the SOCAL and SLIPS samples and had a much higher CAH—the CAH values of PDMS, SOCAL, and SLIPS were 21.4° (*θ*_Adv_ = 116.8 ± 1.5°, *θ*_Reced_ = 95.4 ± 1.3°), 2.5° (*θ*_Adv_ = 102.1 ± 1.1°, *θ*_Reced_ = 99.5 ± 1.8°), and 2.7° (*θ*_Adv_ = 101.2 ± 2.0°, *θ*_Reced_ = 98.5 ± 2.3°), respectively. The similar values of CA and CAH for SOCAL and SLIPS illustrate they can be expected to have similar liquid adhesion and static friction properties. Aerogel was by far the most hydrophobic of the surfaces tested, with a static CA > 150°, and demonstrated an ultralow CAH (<1°) (*θ*_Adv_ = 154.3 ± 6.0°, *θ*_Reced_ = 153.5 ± 6.6°). It can therefore be expected to have low liquid adhesion and low static friction properties. The high CA and ultralow CAH of Aerogel are due to the large volume of air trapped in the porous silica (≅94% of the total volume), resulting in high surface area rough features that are difficult for a droplet to wet.^51^ The high fraction of air at the surface is expected to provide excellent lubrication, and hence extremely low kinetic friction, for droplet motion.

The normal component of the surface tension force from a droplet in contact with a surface *F*_tension_ = *πwγ*_LV_ sin *θ*_e_ is balanced by the normal adhesion force of the surface. Figure 2c provides a comparison of values of measured *πw* sin *θ*_e_ for each of the surfaces tested in this study. As shown in Figure 2c, PDMS, SOCAL, and SLIPS all have similar “sticky” droplet surface adhesion properties (surface adhesion being proportional to sin *θ*_e_ = (sin *θ*_F_ + sin *θ*_B_)/2, as explained in ref (40)), with sin *θ*_e_ = 0.89, 0.97, and 0.96, respectively. In contrast, Aerogel has a relatively “nonsticky” surface adhesion, with sin *θ*_e_ = 0.33, which is about one-third of those for SOCAL and SLIPs,hile the value of *πw* sin *θ*_e_ for Aerogel is about one-fifth of those for SOCAL and SLIPs.

Figure 2d provides a comparison of the kinetic friction, quantified using *μ*_k_/*k*, across the surfaces tested in this study. Despite PDMS, SOCAL, and SLIPS having similar surface adhesion properties, these surfaces have demonstrated markedly different sliding friction characteristics, as seen in Figure 2d. PDMS has the highest kinetic friction of the surfaces tested, with comparable values reported in ref (40). Despite similar CAH, static CA, and surface adhesion between SOCAL and SLIPs, the kinetic friction for the latter is at least five times lower. Aerogel is the most slippery surface with 1 order of magnitude lower kinetic friction than SLIPS. Indeed, for this super-slippery surface, we also observed droplet bouncing during sliding (see Video S1 in the Supporting Information). The AFM images of the solid surfaces (SOCAL, PDMS, and Aerogel) are presented in Figure S1 (Supporting Information). The averaged roughness (Ra) values for SOCAL, PDMS, and Aerogel were 0.24 ± 0.02, 12.97 ± 3.78, and 355.00 ± 146.87 nm, respectively. The surface roughness could affect both CA and CAH. In this study, we believe that the physical nature of the materials plays the key role. For Aerogel, it is air-cushion like surface, which in contact with water droplet and so very low solid surface fraction is the important parameter rather than roughness (as liquid water does not penetrate into the pores). For SLIPs, it is mainly oil atop the PDMS, which is in contact with the water droplet. For SOCAL, it is the uncross-linked PDMS (liquid-like material), covalently bonded to glass, that is in contact with water droplet.

### Droplet Impact Regimes, Ejection, and Bouncing

#### Droplet Impact Regimes

**Figure 3:**
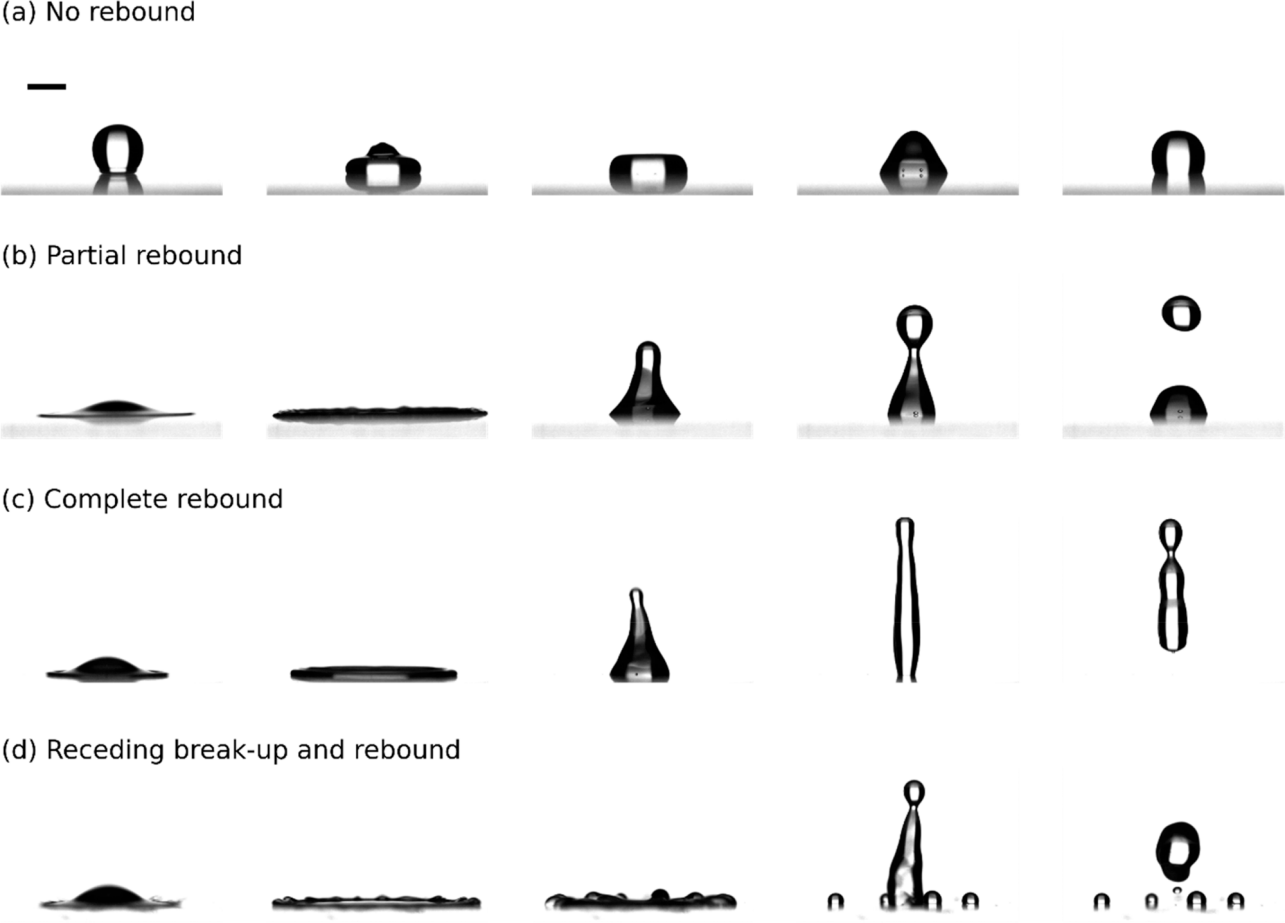
Representative images showing the different impact regimes encountered in this study. (a) The “no rebound” regime where no part of the droplet loses contact with the surface after impact. In this regime, the droplet displays damped oscillation between a changing maxima and minima spread until it eventually comes to rest. (b) The “partial rebound” regime where, after receding from maximum spreading, a portion of the droplet is ejected vertically while the base of the droplet remains pinned to the surface. (c) The “complete rebound” regime where the whole droplet rebounds from the surface following impact. (d) The “receding breakup and rebound” regime where satellite droplets are ejected radially outward while the main drop recedes from the maximum spread and rebounds from the surface. For each regime (a–d), photos from left to right show a progression in time after impact.

**Figure 4:**
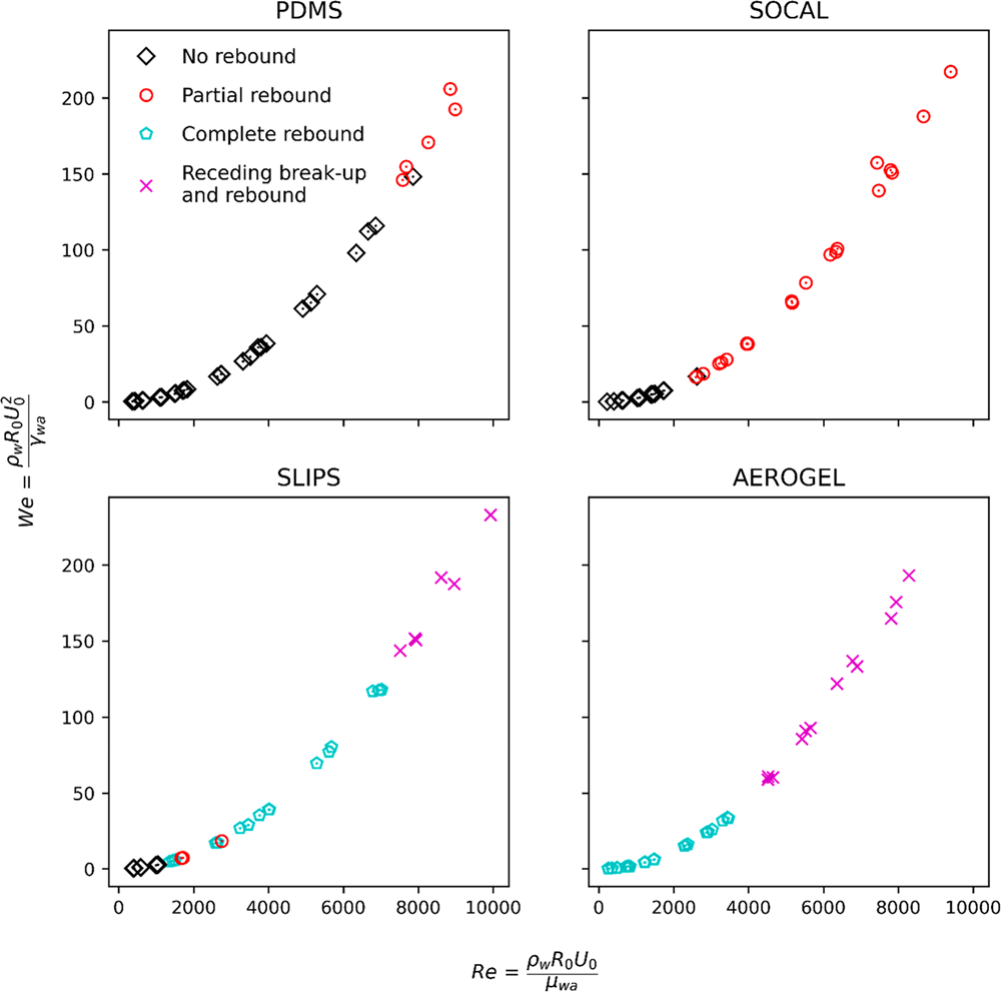
Evolution of impact regime against droplet Weber and Reynold’s numbers for each surface. Each surface is presented in a separate graph to improve clarity (there is significant overlap between curves).

In general, droplet impact behavior followed one of four types/regimes: (1) no rebound, (2) partial rebound, (3) complete rebound, and (4) receding breakup and rebound. Figure 3 provides snapshots illustrating the differences in impact behavior across regimes, and Figure 4 presents the impact regime evolution against *We* and *Re* for each surface. As shown in Figure 4, at low *We* (*We* < 1), all surfaces, except Aerogel, follow the no rebound impact regime, where the energy stored during spreading is insufficient to enable droplet ejection or rebound after retraction. The superhydrophobic (and antiadhesive) nature of Aerogel prevented surface wetting upon impact, and thus, droplets were able to rebound in full at the lowest *We* tested.

For the PDMS and SOCAL surfaces, within the range of *We* (and *Re*) numbers tested in this study, only the first two impact regimes (no rebound and partial rebound) were observed. Partial rebound occurred much earlier on the SOCAL surfaces than on PDMS (*We* ≅ 17 vs *We* ≅ 147), likely due to the SOCAL surfaces having a lower coefficient of kinetic friction (see Figure 2d). For SLIPS, droplet impacts were observed to follow all four regimes, with partial rebound occurring earlier than on the SOCAL surfaces (*We* ≅ 4.7 vs *We* ≅ 17). As *We* (and *Re*) was increased, there was a small overlap of partial and complete rebound regimes observed for SLIPS, possibly due to localized oil loss permitting droplet pinning on impact. This contrast in behavior suggests that the lower kinetic friction of SLIPS for these two surfaces with similar normal adhesion is important (Figure 2d). Only the final two regimes (complete rebound and receding breakup and rebound) were observed for the Aerogel surfaces. Receding breakup and rebound of impacting droplets occurred at much lower *We* (and *Re*) on the Aerogel surfaces than on SLIPS (*We* ≅ 60 vs *We* ≅ 151). This is consistent with the Aerogel having both the lowest kinetic friction (Figure 2d) and the lowest normal adhesion (Figure 2c) of the four surfaces.

#### Droplet Bouncing

Analyzing the droplet height evolution grants insight into the dissipation of energy from the droplet during impact.^52^ To permit cross-comparison of droplet bouncing between tests, a nondimensional droplet height was defined, known in the literature as the bouncing ratio. This bounce ratio, γ, is defined by eq 5 below:
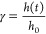
5where *h*(*t*) is the height of the droplet in contact with the surface at time *t* and *h*_0_ is the height of the droplet as it falls toward the surface. In some tests, a secondary droplet is ejected. In such cases, the bouncing ratio of that droplet is labeled as a secondary bounce ratio in the figure legend; likewise, the bounce ratio of tertiary ejected droplets is labeled as tertiary bounce ratios. Where a bounce ratio line breaks (such as with the tertiary droplet ejection for Aerogel in Figure 6a, this is due to the droplet leaving the frame of the video capture.

**Figure 5:**
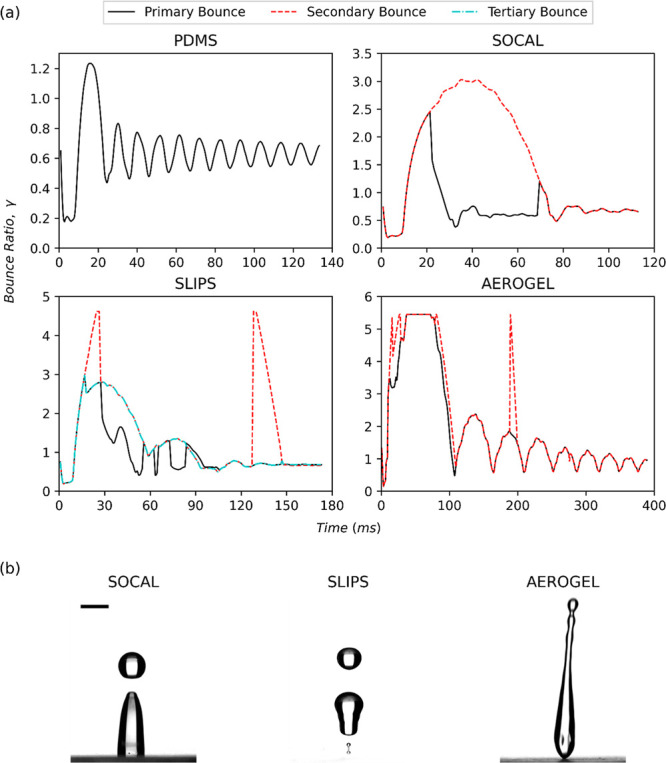
Evolution of the droplet bouncing ratio and several images of droplet ejection at intermediate *We* (30 < *We* < 40). (a) Graphs of the bouncing ratio evolution of droplets impacting on plain PDMS, SLIPS, SOCAL, and Aerogel for intermediate *We*. Droplet ejection occurred on all surfaces except plain PDMS. (b) Droplet ejection images from SLIPS, SOCAL, and Aerogel surfaces. A 2 mm scale bar is provided in the left image. For SOCAL, the secondary droplet curve is shown to separate from the primary curve: this is when the secondary droplet is ejected from the primary droplet.

As is shown in Figure 5, for low *We*, the bouncing ratio evolution for SOCAL and SLIPS follows a similar trend; for plain PDMS, the height of the droplet is much more oscillatory when compared to the other surfaces; and the SLIPS surface in the intermediate *We* number range is the only surface to have both a secondary and tertiary droplet eject. Unlike SOCAL, the ability of SLIPS to eject both a secondary and tertiary droplet at low-intermediate *We* numbers, despite having similar CA and CAH to SOCAL, could be due to its lower kinetic friction permitting droplets to retain more of their initial impact energy following spreading (see Figure 2d). The small surface adhesion forces on Aerogel (see Figure 2c) could cause the lack of tertiary bouncing; for this low-friction surface, momentum transfer within an impacting droplet need not be facilitated by tertiary ejection (like with SLIPS) as no part of the droplet sticks to the surface.

**Figure 6:**
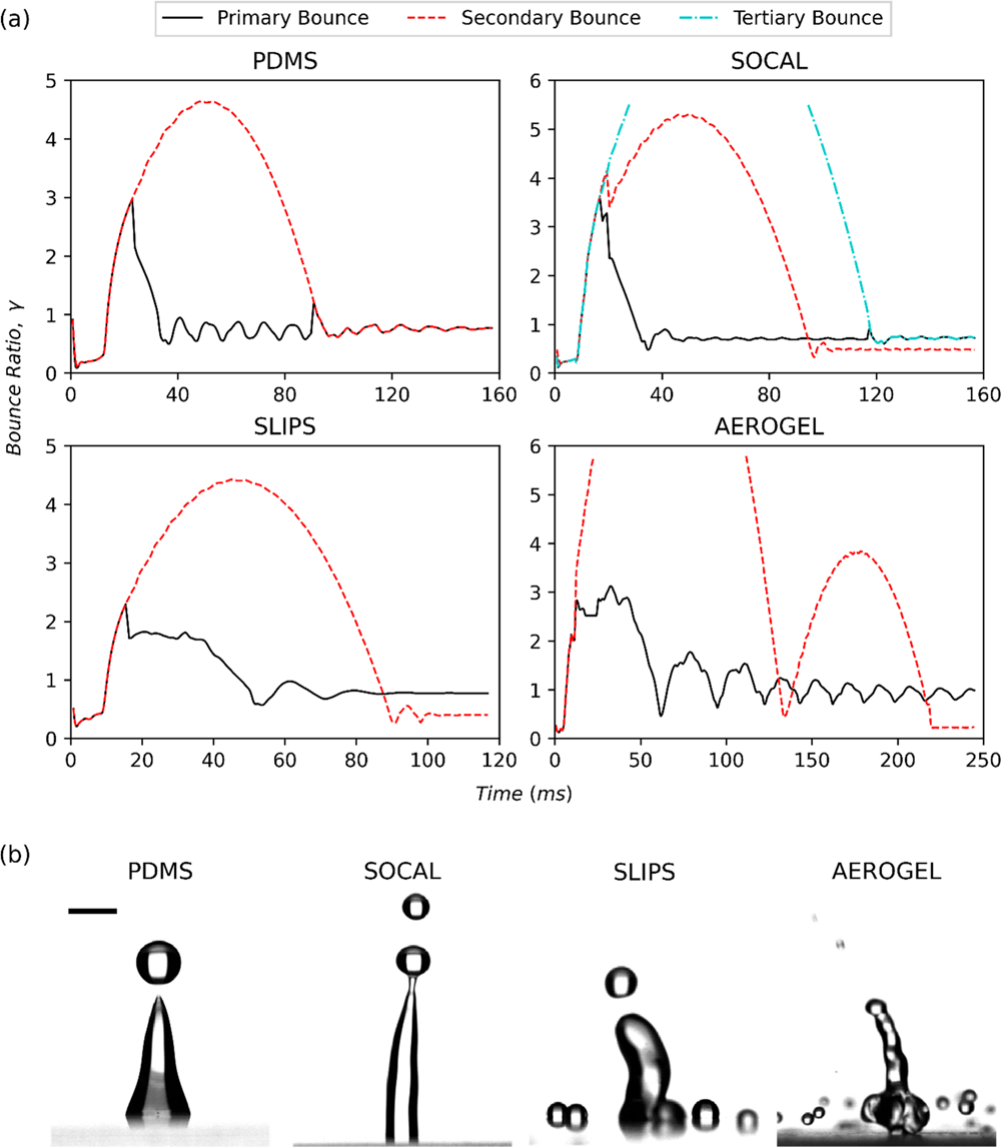
Evolution of the droplet bouncing ratio and several images of droplet ejection at high Weber numbers (150 < *We* < 205). (a) Graphs of the bouncing ratio evolution of droplets impacting on plain PDMS, SLIPS, SOCAL, and Aerogel for high *We*. Droplet ejection occurred on all surfaces including PDMS. (b) Droplet ejection images from SLIPS, SOCAL, and Aerogel surfaces. A 2 mm scale bar is provided in the left image and any breaks in bouncing curves are due to ejected droplets leaving the frame of the captured video.

As shown in Figure 6 for high *We* (*We* > 175), all graphs follow a similar trend wherein a droplet is ejected vertically to a greater height while the base droplet either oscillates vertically adhered to the surface (as is the case for PDMS and SOCAL) or bounces itself several times until it comes to rest. The tertiary bouncing observed in high *We* impacts with SOCAL is likely due to its high normal adhesion forces pinning a portion of the droplet to its surface, like with SLIPS at low-intermediate *We.* This pinning necessitates multiple droplet ejection for full momentum transfer. The reason tertiary ejection occurs at higher *We* values than on SLIPS is likely due to SOCALs’ approximately 5× higher coefficient of kinetic friction causing higher losses in energy throughout impact. It is noted that the Aerogel surface is the only one for which droplets bounced higher at lower *We* than at higher *We*. This is because, at higher *We*, the droplet ejects satellites radially, as seen in Figure 3d, which causes it to lose energy during the spreading process, while for smaller *We* droplets impacting the Aerogel surface have their entire mass rebound upward following retraction (as can be seen from Figure 3b,c).

#### Partial Rebound and Droplet Ejection

In many applications, such as anti-icing, it can be essential to understand what proportion of a droplet stays attached to a surface after impact. A simple measure of this is the proportion of the droplet that leaves the surface after the droplet reaches maximum bounce height. To calculate this for tests where part of the droplet was expelled vertically, the diameters of both the whole droplet and the droplet closest to the surface after ejection were measured in the *x* and *y* directions using ImageJ, and volumes were calculated. By assuming the primary and ejected droplets were ellipsoids, the droplet volumes were calculated using eq 6 below:
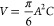
6where the equatorial diameter, *A*, was defined as the diameter in the spreading direction (*x*), and the polar diameter, *C*, was defined to be the height of the droplet in the image plane (*y*).

**Figure 7:**
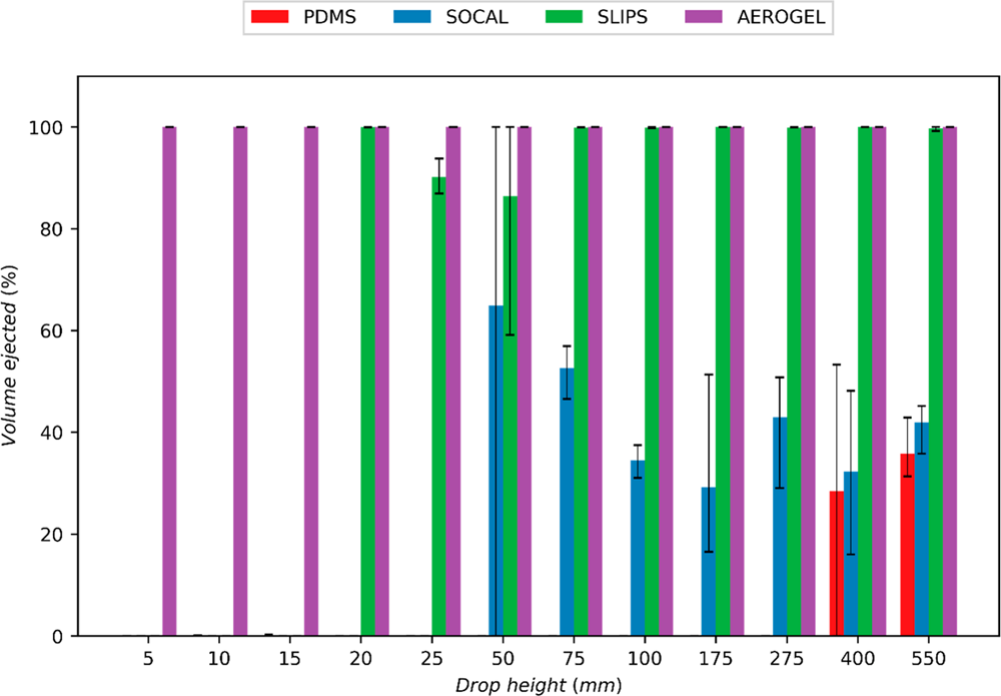
Bar plot showing the proportion of the impacting droplet that is ejected from each surface at each drop height. For clarity, error bars show the max and min measurements instead of the measurements’ standard deviation.

Figure 7 shows how, for most surface types, increasing droplet impact height—and thus, impact speed—increases the proportion of the droplet that leaves the surface. The SOCAL surfaces are the exception to this trend, however, with a large proportion of the droplet remaining on the surface across all heights tested and the fraction of volume ejected having an initial negative trend between 50 and 175 mm drop height. The persistent pinning to SOCAL is likely due to the droplets having insufficient energy to detach from the surface due to losses incurred during spreading and retraction caused by the surfaces’ high coefficient of kinetic friction (see Figure 2d). At the lowest *We* tested (*We* ≅ 0.1), Aerogel was the only surface tested that demonstrated droplet ejection or bouncing, which is likely due to its low normal adhesion force to droplets (see Figure 2c). At higher drop heights (20–25 mm), SLIPS and Aerogel both demonstrated comparable droplet ejection volumes of around 80–100%. At 50 mm drop height, droplet ejection was also recorded on SOCAL samples; however, this was only around 70% of that recorded for Aerogel (see Figure 7). At the highest drop heights, both Aerogel and SLIPS demonstrate considerable (≅100%) droplet ejection. In contrast, the ejection volumes for PDMS and SOCAL were both around 40%, with PDMS having the lowest ejection of the two. The consistently low droplet ejection on PDMS is likely due to its high adhesion forces and high coefficient of kinetic friction (see Figure 2c,d).

One-way ANOVA analysis determined that Aerogel is the only surface with a consistently statistically different volume fraction of droplet ejection compared to all other surfaces (both plain PDMS and SOCAL at low *We*, and plain PDMS at high *We*). As is also shown in Figure 7, Aerogel was the only surface studied that had 100% droplet bouncing across all drop heights tested: This behavior could prove promising for anti-icing applications as no droplet would stay on the surface to be able to form ice crystals.

#### Droplet Contact Time

**Figure 8:**
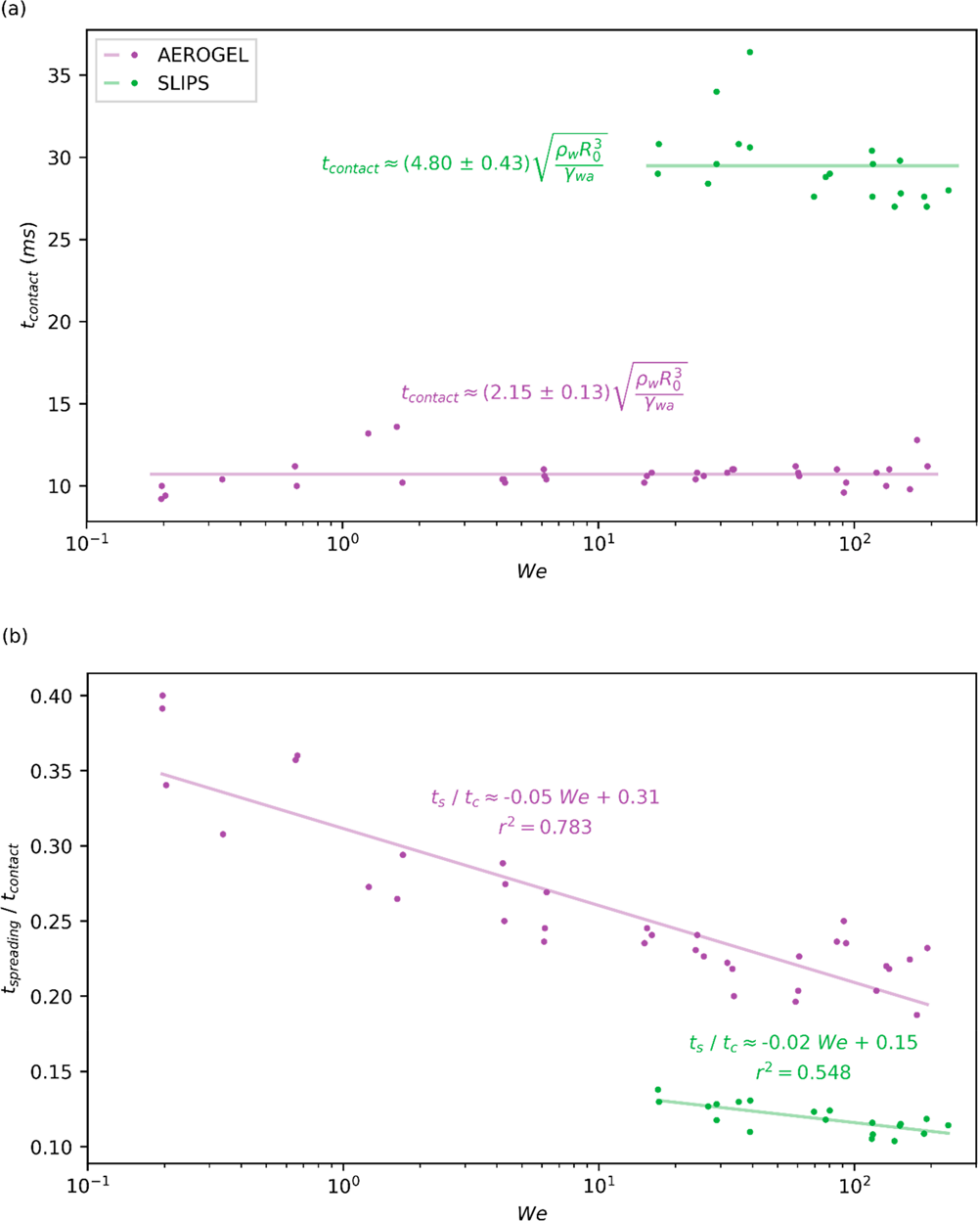
(a) Graph showing the droplet contact time plotted against Weber number. (b) Ratio of spreading time over the contact time for a wide range of *We*. It appears that such a time ratio decreases with the *We* number. The three rebounding SLIPS points shown in Figure 4 (*We* < 10) were not included in fittings as they were present in a region of nonconsistent droplet rebound (see Figure 4).

The contact time between the bouncing droplet and the material surfaces is important as it determines the extent to which mass, momentum, and energy are exchanged on impact. Only Aerogel and SLIPS demonstrated complete rebound (see Figure 4), possibly due to the ultralow adhesion and coefficient of kinetic friction for the Aerogel surface and the low kinetic friction of SLIPS. Therefore, only contact times for these two surfaces were displayed in Figure 8. As shown in Figure 8a, droplet contact times on Aerogel are around  of those on SLIPS at intermediate and high *We*, which is likely due to the superhydrophobic properties of Aerogel, which has a much higher CA and ultralow CAH, as shown in Figure 2a,b. The coefficient of kinetic friction on these surfaces (*μ*_k_/*k* of SLIPS being approximately 7× higher than that of Aerogel) likely also play an important role in the overall droplet contact time on these surfaces as it acts to slow droplet spreading and retraction. The contact times on Aerogel were observed to be independent of *We* and were semiconstant across *We* for SLIPS (one contact time for the inconsistent bouncing at low *We*, and another contact time for higher *We* bouncing). Similarly, Richard et al. reported that the contact time of droplets impacting superhydrophobic solids remained constant across a range of impact velocities, and, by balancing droplet inertia and capillarity, yielded a relationship between droplet properties and the contact time, given in eq 7 below.^53^
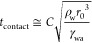
7where ρ_w_ is the density of water, *r*_0_ is the initial droplet radius, *γ*_wa_ is the surface tension at the air–water interface, and *C* is some constant to be found. This relationship was also demonstrated by Guo et al. for petal bounding on a superhydrophobic grooved surface,^54^ where a semiconstant (constant over impact regime) contact time was observed across varying *We*. In this study, it was found that the coefficient, *C*, for Aerogel is around 45% of that for SLIPS at intermediate and high *We*, respectively. Fitting equations and curves of *t*_contact_ are presented in Figure 8a. The short contact time of droplets impacting Aerogel suggests that it has practical importance in applications like anti-icing and self-cleaning, as demonstrated for similar materials in ref (55 and 56).

### Droplet Spreading and Predicting *β*_max_

#### Time-Dependent Droplet Spreading

To quantify the effect of surface characteristics on droplet spreading, a spreading ratio was defined to allow comparative plotting and analysis between tests. This spreading ratio, β is defined by eq 8 below:
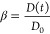
8where *D*(*t*) is the diameter of the droplet in contact with the surface (the diameter of the wetting region) at time *t*, and *D*_0_ is the initial diameter of the droplet as it fell toward the surface.

**Figure 9:**
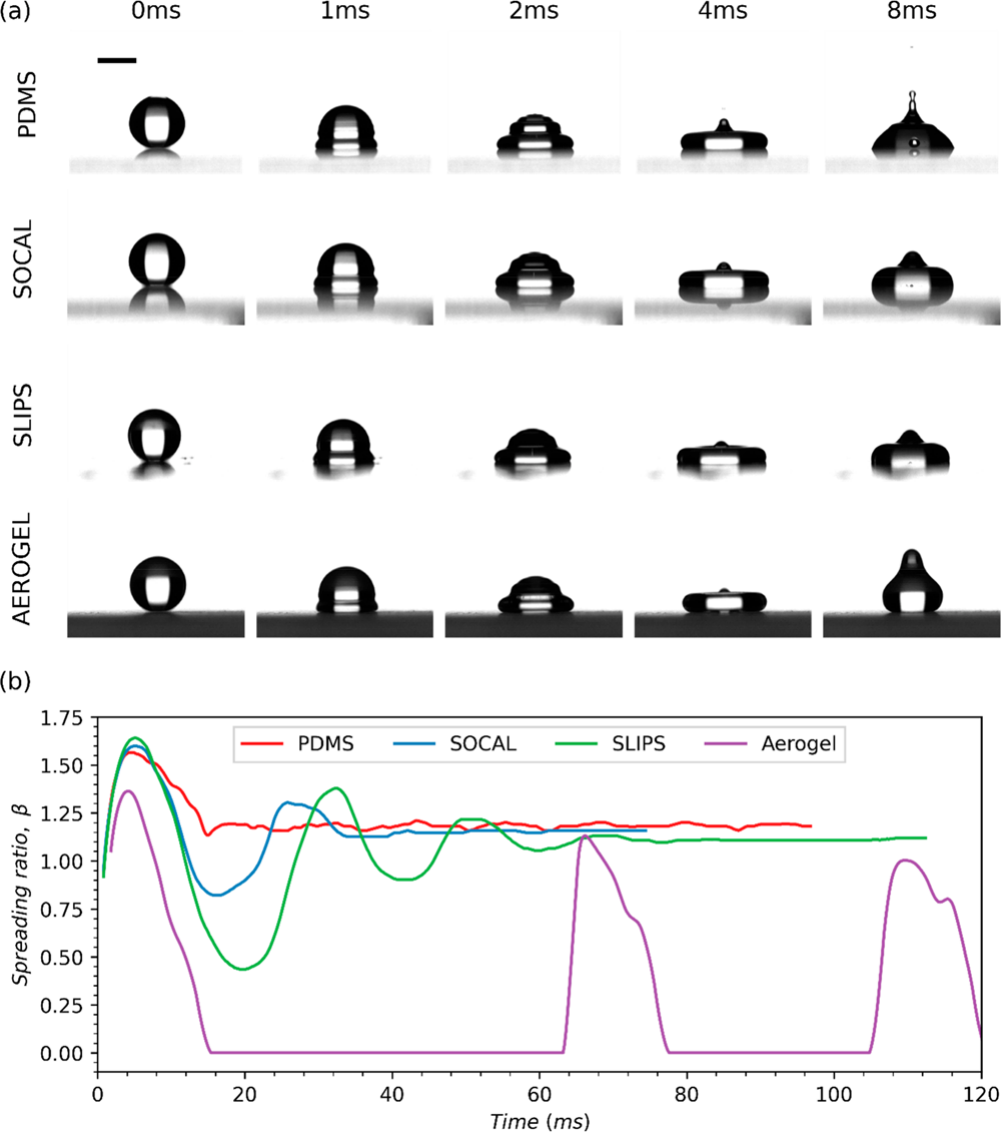
Droplet spreading dynamics at low Weber number (1 < *We* < 4) corresponding to a drop height of 15 mm. (a) Selected snapshots of impacting droplets on each of the surfaces tested in this study (the first four images show droplet spreading, and the final image shows droplet retraction). A 2 mm scale bar is provided in the upper left image. (b) Comparison of the spreading ratio evolutions of droplets impacting each of the four surfaces tested.

Figure 9 presents selected snapshots of droplets impacting each surface and the evolution of the droplet spreading ratio β, against time at low *We* (1 < *We* < 4). At this low *We*, droplets impacting SLIPS, SOCAL, and PDMS followed the no rebound regime (see Figure 4). Thus, the droplet spreading ratio evolution across these surfaces followed a damped oscillation between closing maxima and minima, as shown in Figure 9. Of these three surfaces, SOCAL and SLIPS demonstrate the most similar spreading behavior at this *We*, as low pinning forces on these surfaces did little to damp droplet oscillation, unlike PDMS, where high kinetic friction quickly brought the droplet to rest. SOCAL has higher damping than SLIPS due to higher kinetic friction (as seen in Figure 2d). There is virtually no damping for Aerogel as its kinetic friction is close to zero. Aerogel followed the complete rebound regime at 1 < *We* < 4; hence, as shown in Figure 9, the spreading ratio first increased to a maximum (approximately 1.3), receded to zero at the point of rebound, and remained zero until the droplets’ subsequent contact with the surface. Zero is used for noncontact as, in this study, we have used the diameter of the surface wetted region to define the spreading ratio. During the first spread-detachment cycle, the maximum spreading ratio, β_max_, for PDMS, SLIPS, and SOCAL was approximately 1.6–1.65, which is similar to other superhydrophobic surfaces reported in the literature including other researchers’ SLIPS.^57^ This is approximately 25% higher than the recorded β_max_ value for Aerogel.

**Figure 10:**
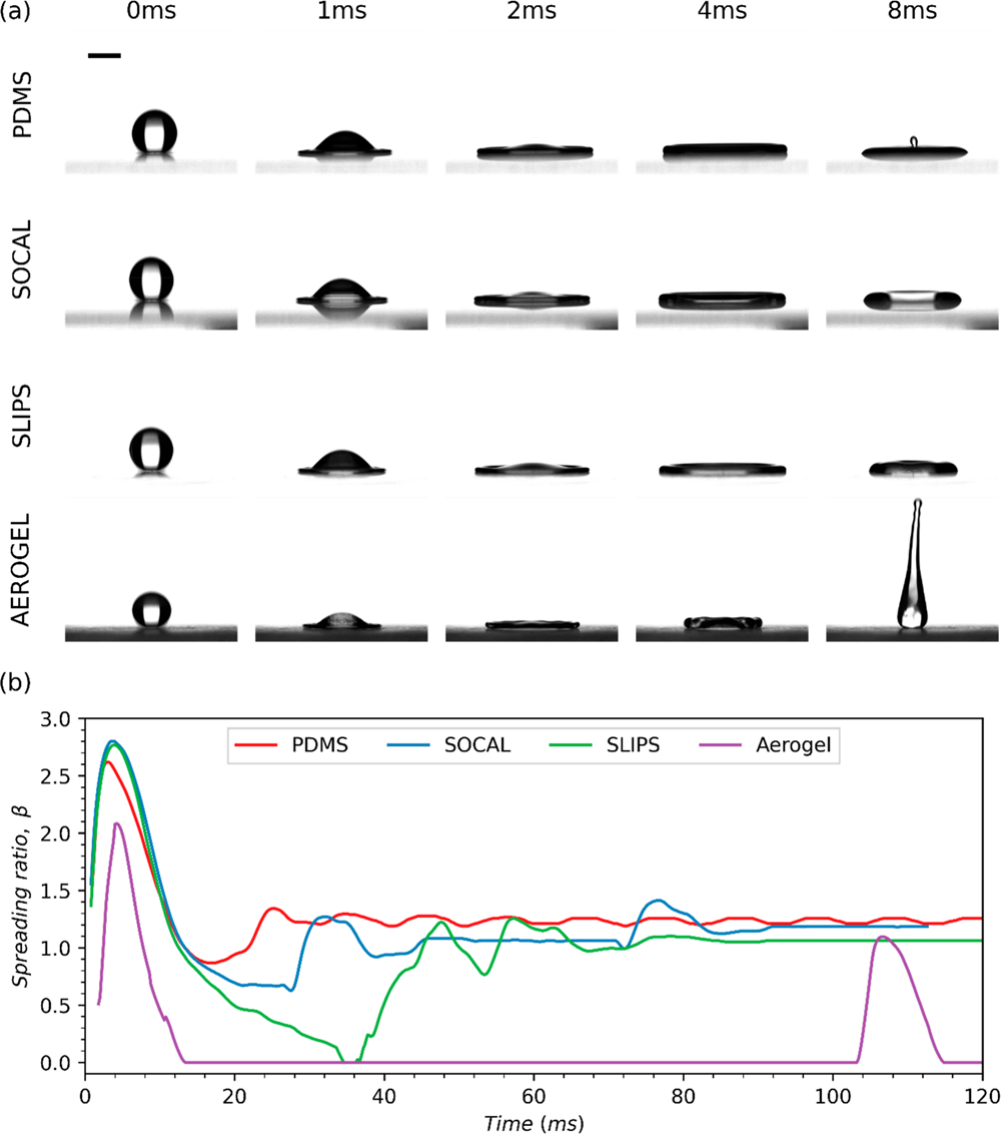
Droplet spreading dynamics at intermediate Weber number (30 < *We* < 40) corresponding to a drop height of 100 mm. (a) Selected snapshots of impacting droplets on each of the surfaces tested in this study (the first three images show droplet spreading, and the final image shows droplet retraction). A 2 mm scale bar is provided in the upper left image. (b) Comparison of the spreading ratio evolutions of droplets impacting each of the four surfaces tested.

As shown in Figure 10a, at higher *We* (30 < *We* < 40), the droplet expands and retracts the quickest when impacting Aerogel, followed by SLIPS. Values of β_max_ increase across all surfaces for greater values of *We*, as seen in Figure 10b. For PDMS, SLIPS, and SOCAL, β_max_ ranges from 2.6 to 2.8 but is only around 2.1 for the Aerogel surface (see Figure 10b). Due to the increased kinetic energy (K.E.) at impact, droplet oscillation following impact on PDMS, SLIPS, and SOCAL is more pronounced in this higher *We* range when compared to the low *We* tests, as can be seen in Figure 10b. Additionally, the increase in K.E. also increases the time between bounces on the Aerogel surface due to the rebounding droplet being propelled higher off the surface.

**Figure 11:**
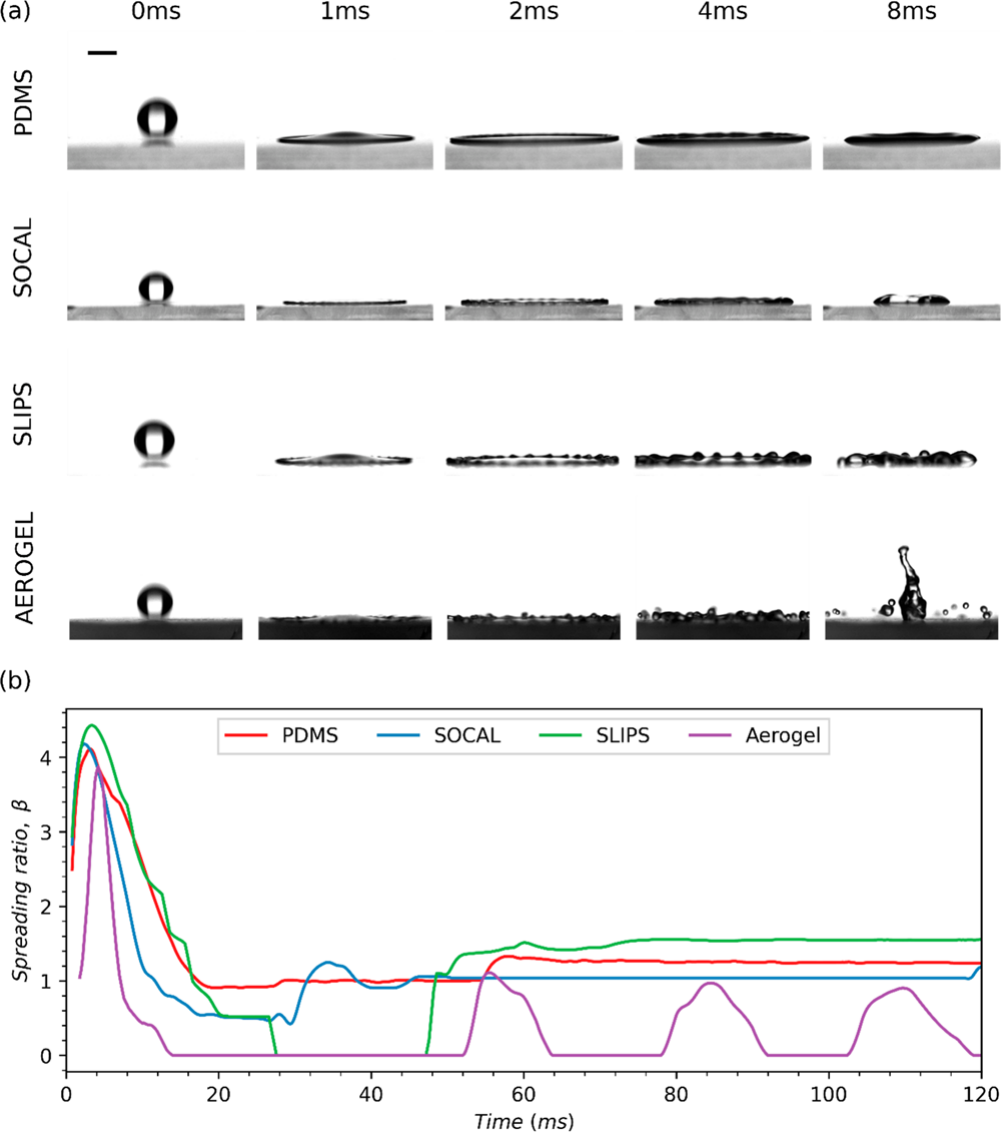
Droplet spreading dynamics at low Weber number (150 < *We* < 250) corresponding to a drop height of 550 mm. (a) Selected snapshots of impacting droplets on each of the surfaces tested in this study (the first four images show droplet spreading, and the final image shows droplet retraction). A 2 mm scale bar is provided in the upper left image. (b) Comparison of the spreading ratio evolutions of droplets impacting each of the four surfaces tested.

As shown in Figure 11a, at much higher *We* (150 < *We* < 205), as with the intermediate range *We* tests, the droplet expands and retracts the quickest when impacting Aerogel, followed by SLIPS. In this high *We* range, receding breakup and rebound was observed for both Aerogel and SLIPS (see Figure 4), while droplets impacting PDMS and SOCAL both followed a partial rebound regime. As is shown in Figure 11, at this *We*, β_max_ increased yet further to 3.8–4.2 across all surfaces, with Aerogel remaining the surface with the lowest maximum spreading ratio. These findings are consistent with those published in the literature for other hydrophobic surfaces such as polytetrafluoroethylene (PTFE) and silicone oil-infused PTFE.^58^

#### Modeling Maximum Spreading Ratio

Analysis of the spreading ratio evolution across different surfaces is not only crucial for practical applications, such as understanding heat transfer during a spray cooling process,^46^ but also allows for the determination and comparison of the maximum spreading ratio, β_max_, which can give insight into the dominant forces acting on the droplet during spreading—such as liquid surface tension and viscous dissipation.^58^ Due to this importance, many theoretical and empirical models have already been developed in the literature to predict the β_max_ values of droplets impacting different surfaces.^58−63^ Sadly, however, despite their success in describing the maximum spreading ratio for some specific materials as reported in the literature, many of these models (eqs S1–S7 in the Supporting Information) provided a generally poor overall β_max_ fitting for our data and sample set, as evidenced in Figure S2 in the Supporting Information.

Of the models tested, the analytical model generated by considering kinetic energy and initial surface energy being converted to new surface energy with viscous energy dissipation^63^ provided the overall best fitting of β_max_ for PDMS, SOCAL, and SLIPs. This model is provided below in eq 9 and considers only the effects of the Weber number (*We*), Reynolds number (*Re*), and the advancing contact angle (*θ*_a_) on the maximum spreading ratio of an impacting droplet.
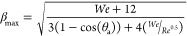
9When deriving this model, it was assumed that viscous dissipation was independent of the material’s surface and that the droplet shape is a flat disk when it is well spread. The model prediction leads to significant discrepancy in the measured results for Aerogel, particularly at mediate and high Weber numbers. This discrepancy is likely due to the viscous dissipation for Aerogel being virtually zero, which is evidenced by complete rebound (see Figure 4), 100% volume rejection (see Figure 7), and zero damping (see Figures 9–11). This low viscous dissipation is due to the extremely low kinetic friction on the Aerogel surface. Therefore, we propose that the viscous term be removed from eq 9 for Aerogel. This is equivalent to regarding the spreading liquid as having a plug flow profile arising from a complete slip boundary condition on the Aerogel.

It is evident that these models overestimate the maximum spreading ratio at low *We* number (*We* < 10) across all our surfaces. This could be due to the fact that the droplet shape was more complex than a flat disk, as observed in the rim-and-dimple side profile shape formed in the initial phase of the dewetting of liquid films from surfaces.^64^ We, therefore, introduce a shape factor (*s*) in the surface energy term at maximum spread in the denominator of eq 9. Our final equations to model the maximum spreading ratio are therefore,
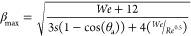
10awhen viscous dissipation occurs (i.e., SLIPS, SOCAL, and PDMS); and
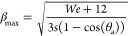
10bwhen viscous dissipation is absent (i.e., super(hydrophobic) Aerogel).

**Figure 12:**
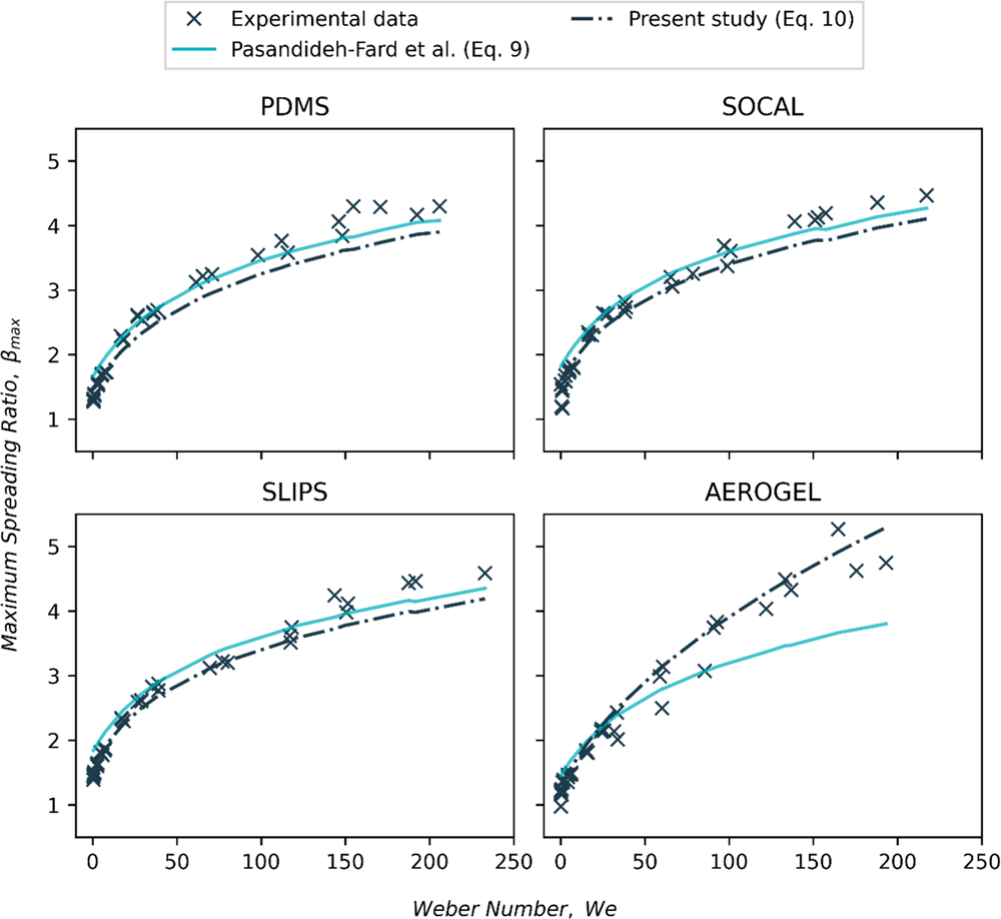
Comparison of β_max_ fittings using the model derived by Pasandideh-Fard et al. (eq 9)^63^ and present study (eqs 10a and 10b).

In this study, an empirical shape factor *s* = 1.28 was found to be applicable to all four surfaces; this was calculated by curve fitting using a custom python script. Figure 12 shows the data from the maximum spreading ratio of droplets impacting on the four surfaces is well-described by eqs 10a and 10b. The ability to fit data for PDMS, SOCAL, and SLIPS reasonably well using eqs 10a and 10b, with just the viscous dissipation, suggests differences in their kinetic friction primarily influence the impact and rebound experiments’ dewetting (retraction) phase.

## Conclusions

In this study, we have carefully examined droplet interactions on three promising liquid repelling surfaces with different liquid adhesion and kinetic friction properties: a liquid-infused surface (PDMS infused with silicone oil to give a SLIP surface), a liquid-like solid surface (SOCAL), and an air-cushion-like surface (superhydrophobic Aerogel). SOCAL and SLIPS have almost identical characteristics for static (or quasi-static) interactions with a droplet, as characterized by contact angle and contact angle hysteresis. Despite this, these surfaces exhibit a distinctly different physical fingerprint in their dynamic interaction with droplets during sliding and impact, as evidenced by their droplet bouncing types, bouncing ratios, spreading dynamics, and contact times during impact measured in this study. This difference could be due to their previously reported differences in the kinetic (dynamic) friction.^36^ Aerogel has the highest contact angle among all the surfaces tested and has an ultralow contact angle hysteresis (<0.7°) and kinetic friction (*μ*_k_/*k* ≅ 0.0041), which is due to an ultrahigh air density (≅ 94% volume). It is therefore an anti-adhesive surface with significant lubrication for droplet motion. As such, Aerogel demonstrated complete rebound at a very low Weber number (∼1) with 100% ejection volume and the shortest contact time among all the surfaces studied here. Aerogel also demonstrated no damping effects during spreading process with negligible viscous dissipation. These exceptional characteristics will make Aerogel an ideal surface for liquid repellence, anti-icing, and many other important industrial applications, followed by SLIPS, which exhibits similar behavior but at a higher Weber number. We have also proposed an improved droplet spreading model for materials with non-negligible and negligible viscous dissipation. This model provided good fitting to all four surfaces at a wide range of Weber numbers, which was not achieved by other models. Finally, the emphasis we have placed on understanding the relationship between liquid adhesion normal to a surface and the difference between static and kinetic liquid friction along the surface has important implications for processes such as, inkjet printing, spray coating, heat transfer efficiency in spray/droplet cooling applications, and bloodstain formation in forensic science.

## Data Availability

All the data that support the findings of this study are present in the paper and the Supporting Information. Additional data related to this paper may be requested from the authors.

## Supporting Information Available

Figures of AFM images of all the solid surfaces and graphs showing comparisons between the predictions of β_max_ and discussions of modelling maximum spreading ratio (PDF)
Video of the bouncing associated with sliding for Aerogel surface (MP4)

## Abbreviations Used

SLIPS: liquid-infused porous surface
PDMS: polydimethylsiloxane
SOCAL: slippery omniphobic covalently attached liquid-like
CA: contact angle
CAH: contact angle hysteresis
