# Correction to “Dynamics of Droplets Impacting on Aerogel, Liquid Infused, and Liquid-Like Solid Surfaces”

**DOI:** 10.1021/acsami.3c16610

**Published:** 2023-11-16

**Authors:** Jack Dawson, Samual Coaster, Rui Han, Johannes Gausden, Hongzhong Liu, Glen McHale, Jinju Chen

In the original version of this article, the droplet radius was conflated with the droplet diameter when processing the raw data causing a factor of 2 error in the Weber number (*We*) in some text and figure captions and in Figures 4, 8, 12 and Figure S2. Additionally, a formatting error occurred in eq 4. The corrected text and figures are described below. This correction does not alter the major conclusions of this work.

(1) In the abstract, all the presented Weber number values and ranges should be doubled. A bullet-pointed list of these corrections is provided below:

- “··· rebound can start at a very low Weber (*We*) number (∼1).” should instead read “··· rebound can start at a very low Weber (*We*) number (∼2).”
- “··· complete rebound only occurs at a much higher *We* number (>5).” should instead read “··· complete rebound only occurs at a much higher *We* number (>10).”
- “··· complete rebound was not observed, even for a *We* as high as 200.” should instead read “··· complete rebound was not observed, even for a *We* as high as 400.”
- “··· was only observed consistently when the *We* was above 5–10.” should instead read “··· was only observed consistently when the *We* was above 10–20.”
- “··· not observed even at the highest *We* number tested here (∼200).” should instead read “··· not observed even at the highest *We* number tested here (∼400).”

(2) In eq 4, the *R* should be removed. The corrected eq 4 is
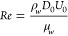
4

(3) In Figure 4, the *R*_0_ symbol should be replaced with a *D*_0_ symbol in the axis labels. Additionally, the *y*-axis values of all plots should be scaled by a factor of 2 to reflect the new Weber number range. The corrected figure and original figure caption are given below.

**Figure 4:**
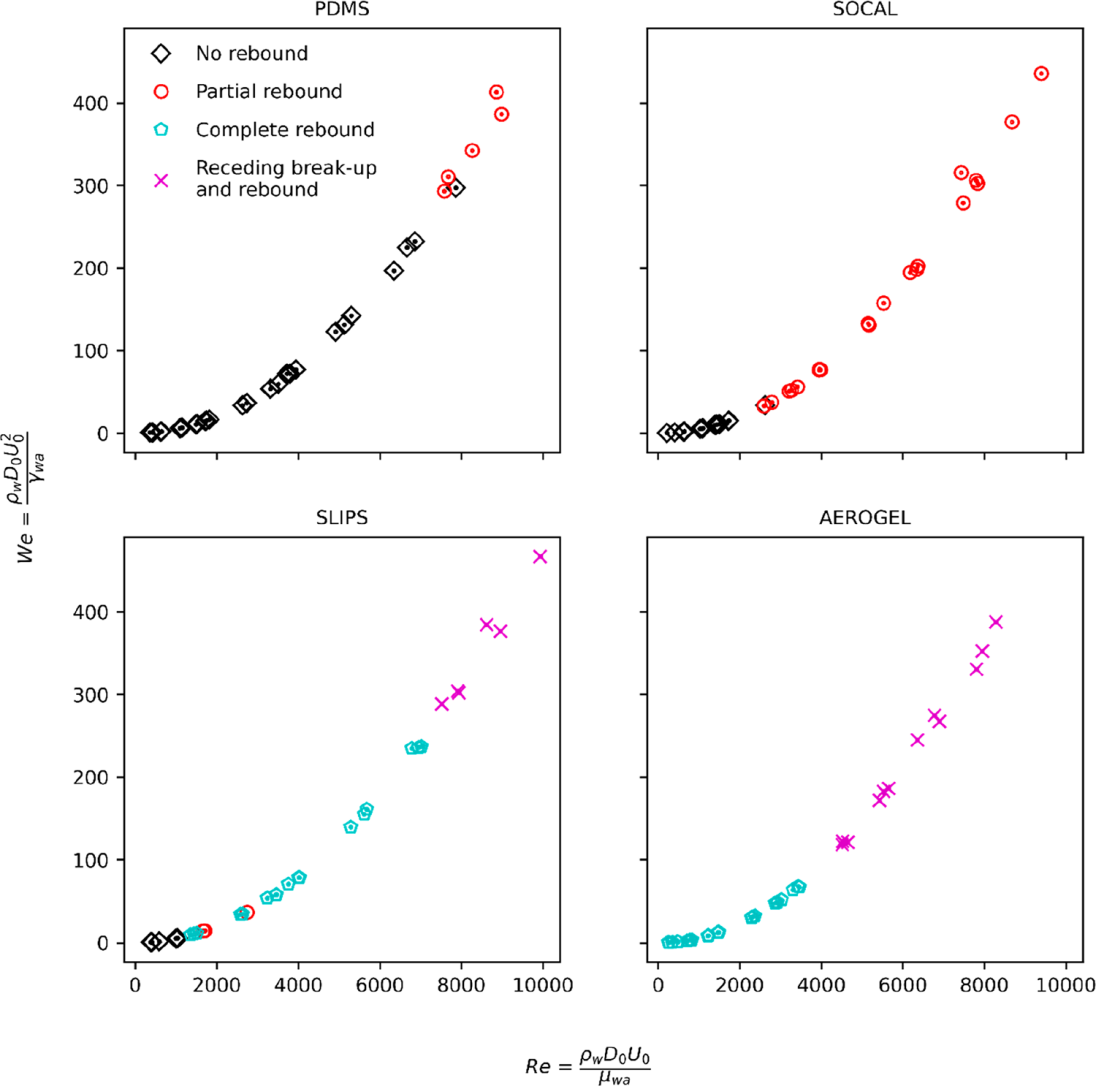
Evolution of impact regime against droplet Weber and Reynold’s numbers for each surface. Each surface is presented in a separate graph to improve clarity (there is significant overlap between curves).

(4) In the “**Droplet Impact Regimes, Ejection, and Bouncing.***Droplet Impact Regimes*” section, the Weber number values and ranges presented should all be doubled. A bullet-pointed list of these corrections is provided below:

- “As shown in Figure 4, at low *We* (*We* < 1), ...” should instead read “As shown in Figure 4, at low *We* (*We* < 2), ...”
- “Partial rebound occurred much earlier on the SOCAL surfaces than on PDMS (*We* ≅ 17 vs *We* ≅ 147), ...” should instead read “Partial rebound occurred much earlier on the SOCAL surfaces than on PDMS (*We* ≅ 34 vs *We* ≅ 294), ...”
- “than on the SOCAL surfaces (*We* ≅ 4.7 vs *We* ≅ 17).” should instead read “··· than on the SOCAL surfaces (*We* ≅ 9.4 vs *We* ≅ 34).”
- “on the Aerogel surfaces than on SLIPS (*We* ≅ 60 vs *We* ≅ 151).” should instead read “··· on the Aerogel surfaces than on SLIPS (*We* ≅ 120 vs *We* ≅ 302).”

(5) In the caption of Figure 5, the Weber value ranges should be doubled. The corrected caption is “**Figure 5.** Evolution of the droplet bouncing ratio and several images of droplet ejection at intermediate *We* (60 < *We* < 80). (a) Graphs of the bouncing ratio evolution of droplets impacting on plain PDMS, SLIPS, SOCAL, and Aerogel for intermediate *We*. Droplet ejection occurred on all surfaces except plain PDMS. (b) Droplet ejection images from SLIPS, SOCAL, and Aerogel surfaces. A 2 mm scale bar is provided in the left image. For SOCAL, the secondary droplet curve is shown to separate from the primary curve: this is when the secondary droplet is ejected from the primary droplet.”

6) In the “**Droplet Impact Regimes, Ejection, and Bouncing.***Droplet Bouncing”* section, above Figure 6, in the paragraph starting “As shown in Figure 6 ...”, the stated Weber value should be doubled from 175 to 350. This paragraph should open “As shown in Figure 6 for high *We* (*We* > 350), ...”.

(7) The Weber value range presented in the caption of Figure 6 should be doubled. The corrected caption is **Figure 6.** Evolution of the droplet bouncing ratio and several images of droplet ejection at high Weber numbers (300 < *We* < 410). (a) Graphs of the bouncing ratio evolution of droplets impacting on plain PDMS, SLIPS, SOCAL, and Aerogel for high *We*. Droplet ejection occurred on all surfaces including PDMS. (b) Droplet ejection images from SLIPS, SOCAL, and Aerogel surfaces. A 2 mm scale bar is provided in the left image and any breaks in bouncing curves are due to ejected droplets leaving the frame of the captured video.”

(8) In the “**Droplet Impact Regimes, Ejection, and Bouncing.***Partial Rebound and Droplet Ejection*” section, the text displayed after Figure 7 reading “At the lowest *We* tested (*We* ≅ 0.1), Aerogel was the only surface···” should have its stated Weber number value doubled to 0.2. The corrected sentence should read “At the lowest *We* tested (*We* ≅ 0.2), Aerogel was the only surface···”

(9) In Figure 8, the *x*-axis values should be doubled and the caption updated to state the correct Weber range. The updated figure and caption are given below.

**Figure 8:**
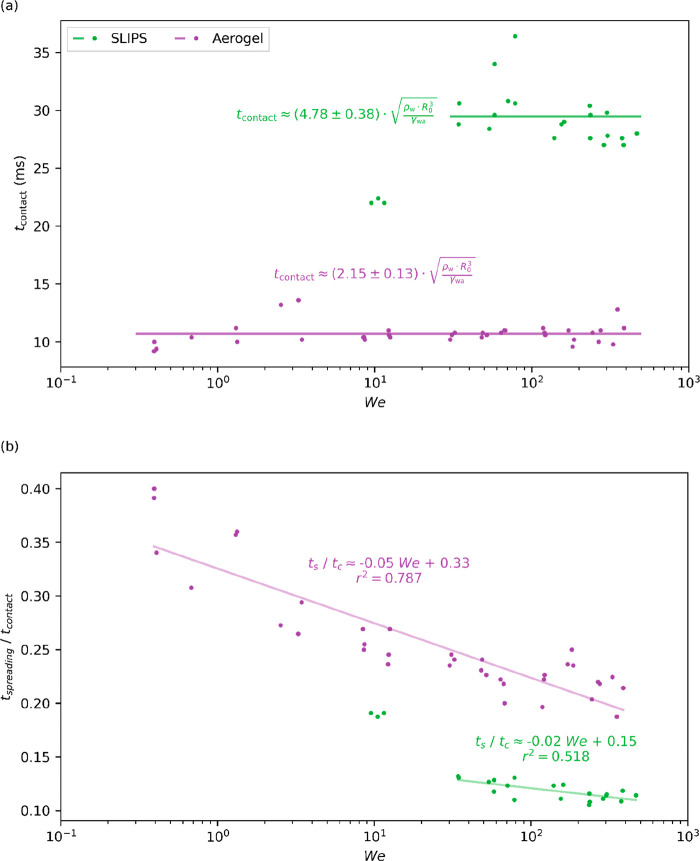
(a) Graph showing the droplet contact time plotted against Weber number. (b) Ratio of spreading time over the contact time for a wide range of We. It appears that such a time ratio decreases with the We number. The three rebounding SLIPS points shown in Figure 4 (*We* < 20) were not included in fittings as they were present in a region of nonconsistent droplet rebound (see Figure 4).

(10) In the “**Droplet Spreading and Predicting β**_***max***_. *Time-Dependent Droplet Spreading*” section, all Weber values presented in the text should be doubled. A bullet-pointed list of these corrections is provided below:

- “droplet spreading ratio β, against time at low *We* (1 < *We* < 4).” should instead read “··· droplet spreading ratio β, against time at low *We* (2 < *We* < 8).”
- “Aerogel followed the complete rebound regime at 1 < *We* < 4; ···” should instead read “Aerogel followed the complete rebound regime at 2 < *We* < 8; ···”.
- “As shown in Figure 10a, at higher *We* (30 < *We* < 40), ...” should instead read “As shown in Figure 10a, at higher *We* (60 < *We* < 80), ...”
- “As shown in Figure 11a, at much higher *We* (150 < *We* < 205), ...” should instead read “As shown in Figure 11a, at much higher *We* (300 < *We* < 410), ...”

(11) In the caption of Figure 9, the Weber value range should be doubled. The corrected caption is “**Figure 9.** Droplet spreading dynamics at low Weber number (2 < *We* < 8) corresponding to a drop height of 15 mm. (a) Selected snapshots of impacting droplets on each of the surfaces tested in this study (the first four images show droplet spreading, and the final image shows droplet retraction). A 2 mm scale bar is provided in the upper left image. (b) Comparison of the spreading ratio evolutions of droplets impacting each of the four surfaces tested.”

(12) In the caption of Figure 10, the Weber value range should be doubled. The corrected caption is “**Figure 10.** Droplet spreading dynamics at intermediate Weber number (60 < *We* < 80) corresponding to a drop height of 100 mm.(a) Selected snapshots of impacting droplets on each of the surfaces tested in this study (the first three images show droplet spreading, and the final image shows droplet retraction). A 2 mm scale bar is provided in the upper left image. (b) Comparison of the spreading ratio evolutions of droplets impacting each of the four surfaces tested.”

(13) In the caption of Figure 11, the Weber value range should be doubled. The corrected caption is “**Figure 11.** Droplet spreading dynamics at low Weber number (300 < *We* < 500) corresponding to a drop height of 550 mm. (a) Selected snapshots of impacting droplets on each of the surfaces tested in this study (the first four images show droplet spreading, and the final image shows droplet retraction). A 2 mm scale bar is provided in the upper left image. (b) Comparison of the spreading ratio evolutions of droplets impacting each of the four surfaces tested.”

(14) In the “**Droplet Spreading and Predicting β**_***max***_. *Modeling Maximum Spreading Ratio.*” section, the value of the empirical shape factor, *s*, should be changed from 1.28 to 2.12. The corrected text is “In this study, an empirical shape factor *s* = 2.12 was found···”

(15) In the “**Droplet Spreading and Predicting β**_***max***_. *Modeling Maximum Spreading Ratio.*” section, the Weber value presented in text at the start of the paragraph above eq 10a should be doubled. The corrected sentence is “It is evident that these models overestimate the maximum spreading ratio at low *We* number (*We* < 20) across all our surfaces”.

(16) In Figure 12, the Weber number range should be doubled. The corrected figure and original figure caption are given below.

**Figure 12:**
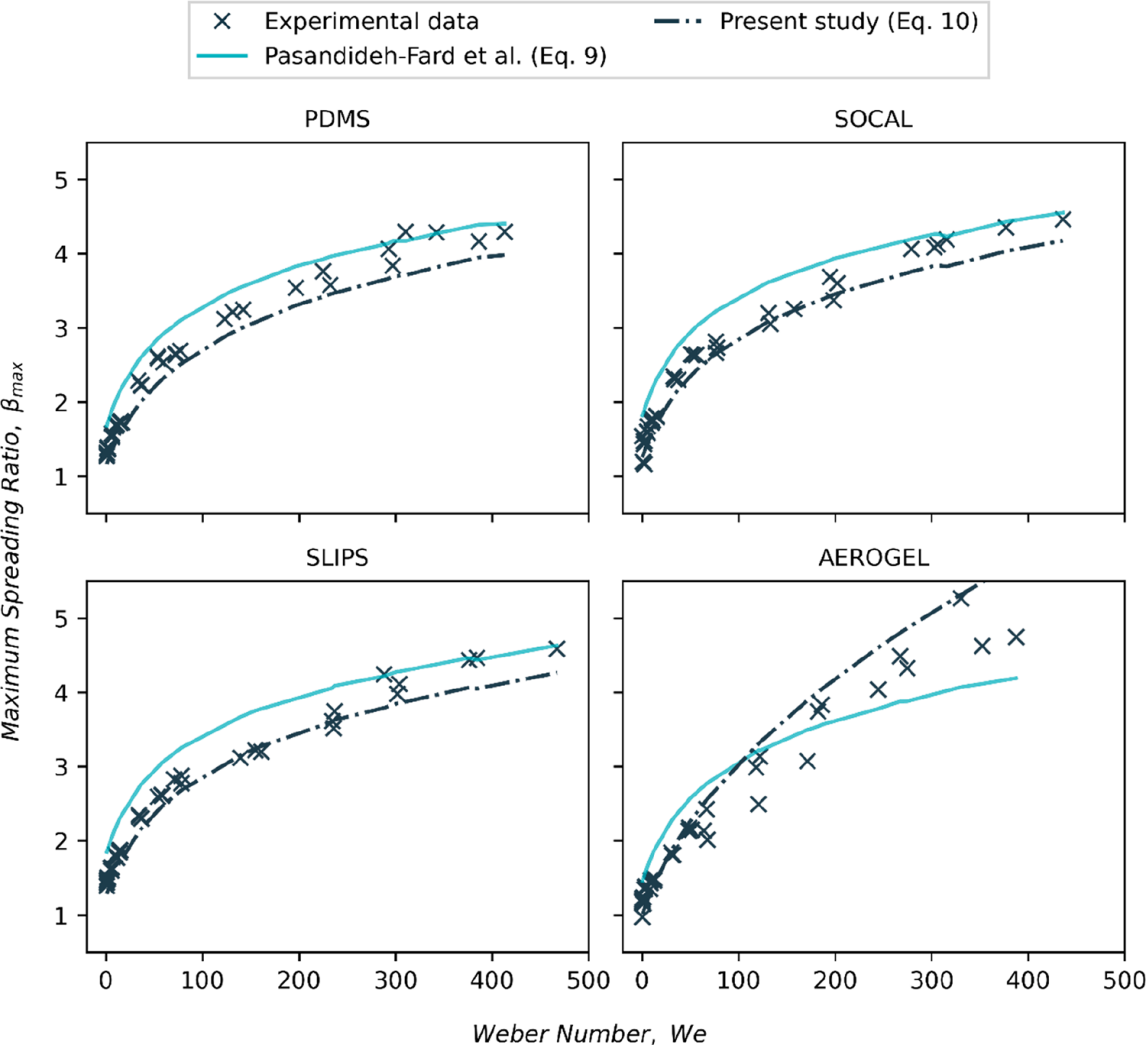
Comparison of β_*max*_ fittings using the model derived by Pasandideh-Fard et al. (eq 9)^63^ and present study (eqs 10a and 10b).

(17) In the “**CONCLUSIONS**” section, the Weber value presented in the text should be doubled. The corrected sentence is “As such, Aerogel demonstrated complete rebound at a very low Weber number (∼2) with 100% ejection volume and the shortest contact time among all the surfaces studied here.”

(18) In the “**Supporting Information**”, Weber number values and ranges shown in text should be doubled. A bullet-pointed list of these corrections is provided below:

“overestimates β_*max*_ when *We* falls below 125.” should instead read “··· overestimates β_*max*_ when We falls below 250.”.
“Roisman’s model only provides a decent fit of β_*max*_ for 25 ≤ *We* ≤ 50.” should instead read “··· Roisman’s model only provides a decent fit of β_*max*_ for 50 ≤ *We* ≤ 100.”.
“··· overestimate of β_*max*_ at low *We* (*We* < 50) then severely underestimates β_*max*_ from intermediate-high *We* (*We* > 100).” should instead read “··· overestimate of β_*max*_ at low *We* (*We* < 100) then severely underestimates β_*max*_ from intermediate-high We (*We* > 200).”.
“··· it underestimates β_*max*_ at high *We* (*We* > 100) for Aerogel.” should instead read “··· it underestimates β_*max*_ at high *We* (*We* > 200) for Aerogel.”.
“Asai et al.’s model underestimates β_*max*_ on Aerogel when *We* is above 100.” should instead read “Asai et al.’s model underestimates β_*max*_ on Aerogel when *We* is above 200.”

(19) In the “**Supporting Information**”, Figure S2 should be updated to display points plot using the new Weber number range. The corrected figure and original figure caption are given below.

**Figure S2:**
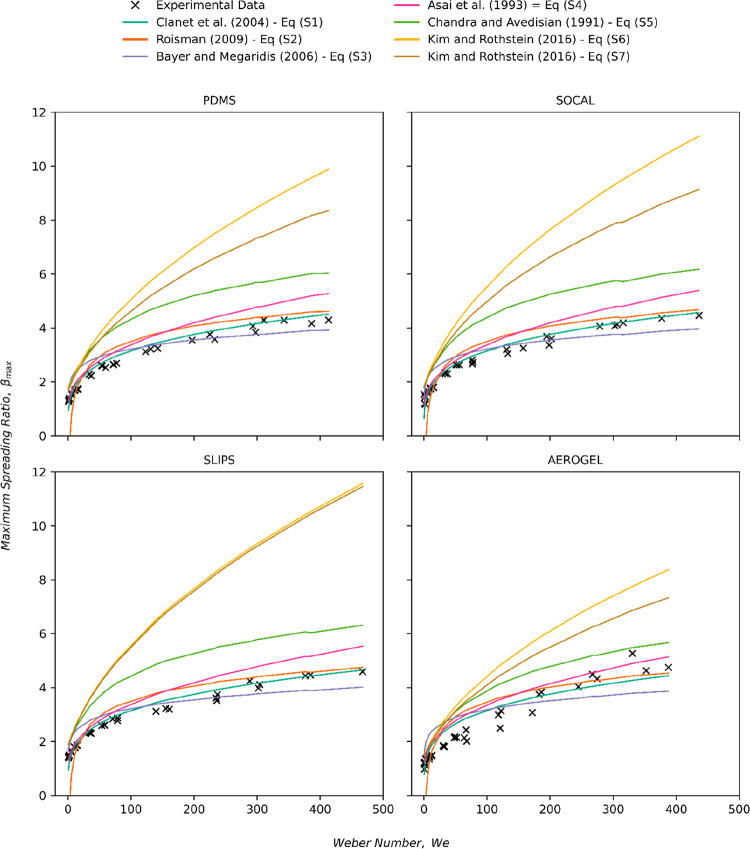
Graphs showing comparisons between the predictions of β_*max*_ made by the models described in eqs S1–S7 and the experimental results for β_*max*_ collected across the four different surfaces tested in this study.

