# Supplementary material for: Dynamics of Droplets Impacting on Aerogel, Liquid Infused, and Liquid-Like Solid Surfaces
Source: ACS Appl Mater Interfaces. 2022 Dec 29;15(1):2301–12. doi: 10.1021/acsami.2c14483 (PMC9837784; doi:10.1021/acsami.2c14483)
Supplement: Supplementary file 1 — am2c14483_si_001.pdf [file am2c14483_si_001.pdf]

Supporting Information

Dynamics of Droplets Impacting on Aerogel, Liquid Infused, and Liquid-Like Solid Surfaces

Jack Dawson [1], Samual Coaster [1], Rui Han[1], Johannes Gausden [1], Hongzhong Liu[2], Glen McHale[3], Jinju Chen [1]*

1 School of Engineering, Newcastle University, Newcastle Upon Tyne, NE1 7RU, UK.

2 School of Mechanical Engineering, Xi'an Jiaotong University, Xi'an 710054, China

3 School of Engineering, Institute for Multiscale Thermofluids, The University of Edinburgh, Edinburgh, EH9 3FB, UK.

* Corresponding author. Email: Jinju.chen@ncl.ac.uk

Number of Pages: 5

The AFM images of all the solid surfaces.

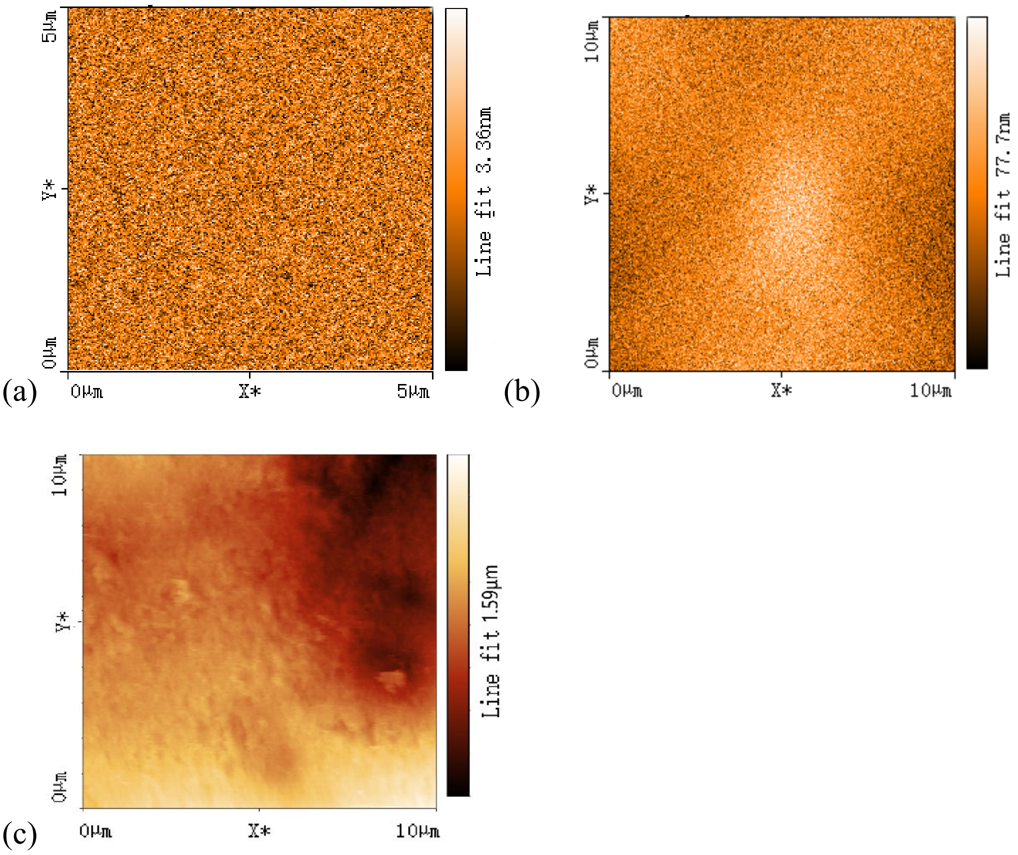

Figure S1. AFM images of (a) SOCAL, (b) PDMS and (c) aerogel.

Modelling Maximum Spreading Ratio – Comparisons across other models

Several of the key equations used to predict β_{max} outlined in the literature, which are used in this study to compare to our results, are provided in this section.

The first of the models used to compare our results to is that proposed by Clanet et al. [1]. This is an empirical model relating β_{max} to We by equation (S1) below:

$$\beta_{max} = \begin{cases} We^{0.25}, & We/_{Re^{0.8}} < 1 \text{ (inviscid)} \\ Re^{0.2}, & We/_{Re^{0.8}} \geq 1 \end{cases} \tag{S1}$$

The semiempirical model derived by Roisman [2] was also used for comparison. This is provided in equation (S2) below:

$$\beta_{max} = 0.87Re^{0.2} - 0.4\, Re^{0.4}\, We^{-0.5} \tag{S2}$$

The empirical model Bayer and Megaridis [3] derived by fitting a regression line to droplet spreading data collected on impacts with flat steel surfaces was also used, and iss provided in equation (S3) below:

$$\beta_{max} = 0.72(Re\, We^{0.5})^{0.14} \tag{S3}$$

An empirical model derived by Asai et al. (1993) [4] for ink jetting on paper is provided in equation (S4) below:

$$\beta_{max} = 1 + 0.48We^{0.5}\exp\,(-1.48\, We^{0.22}\, Re^{-0.21}) \tag{S4}$$

Chandra and Avedisian derived a quartic equation to relate expected β_{max} to We, Re, and the static contact angle of the material [5]. This is provided in equation (S4) below and can be solved to get β_{max}:

$$\frac{3We}{2Re}{\beta_{max}}^4 + \left(1 - cos(\theta_{static})\right){\beta_{max}}^2 - \left(\frac{We}{3} + 4\right) = 0 \tag{S5}$$

Kim and Rothstein derived an expression for β_{max} through conservation of kinetic and interfacial energy [6]. This is provided in equation (S6) below:

$$\beta_{max} = \sqrt{\frac{We + 12}{3\left(1 - \cos\,(\theta_a)\right)}} \tag{S6}$$

Kim and Rothstein also derived an expression for β_{max} on surfaces with a viscous oil layer, such as with SLIPS or LIS. This takes into account the droplet geometry, viscosity ratio

between droplet and viscous surface phase, and the Reynolds and Capillary numbers of the droplet [6]. This expression is provided in equation (S7) below:

$$\beta_{max} = \sqrt{\frac{Re + \frac{12}{Ca}}{12\pi\left[1 + \left(\frac{t}{h}\right)\left(\frac{\mu_w}{\mu_o}\right)\right]^{-1} + \frac{3(1-\cos(\theta_a))}{Ca}}} = \sqrt{\frac{We + 12}{12\, Ca\, \pi\left[1 + \left(\frac{t}{h}\right)\left(\frac{\mu_w}{\mu_o}\right)\right]^{-1} + 3(1-\cos(\theta_a))}} \tag{S7}$$

where $Ca = \frac{\mu_w U_0}{\gamma_{wa}}$ is the capillary number, t is the oil film thickness, h is the thickness of the droplet at maximum spread $\left(h = \frac{2D_0^3}{3D_{max}^2}\right)$, μ_o is the lubricant viscosity (0.93 mPa.s in this study), and θ_a is the advancing contact angle.

Despite their success in describing the maximum spreading ratio for some specific materials reported in the literature, the models (equations S1-S7) summarized here provided a generally poor overall β_{max} fitting for our data and sample set. A comparison between these models (equation S1-S7) and experimental results across all four different surfaces is displayed in Fig. S2. As shown in Fig. S1, for PDMS, SOCAL, and SLIPS, the models proposed by Clanet et al., Chandra and Avedisian, and Kim and Rothstein consistently severely overestimate β_{max}. Of the models proposed by Kim and Rothstein, the model described in equation (S6) overestimates values of β_{max} for Aerogel; whereas the LIS model described in equation (S7) provides a decent fit for intermediate values of We. For Aerogel, the model proposed by Chandra and Avedisian (equation (S5)) provides a decent fit at high We; however, slightly overestimates β_{max} when We falls below 125. It is interesting to note that the LIS model proposed by Kim and Rothstein (equation (S7)) overestimates β_{max} by a lesser degree than the model described in equation (S6) across all samples, including SLIPS. Roisman's model (Eq. (S2)) severely underestimates β_{max} at low We across all surfaces; however, it provides a reasonable approximation at mid-high We for PDMS, SOCAL, and SLIPS. For Aerogel, Roisman's model only provides a decent fit of β_{max} for $25 \leq We \leq 50$. Across all surfaces, the model proposed by Bayer and Megaridis (equation (S3)) provides a moderate overestimate of β_{max} at low We ($We < 50$), then severely underestimates β_{max} from intermediate-high We ($We > 100$). For PDMS, SOCAL, and SLIPS, the empirical model proposed by Asai et al. (equation. (S4)) fits β_{max} reasonably well across a range of We;

however, it underestimates β_{max} at high We ($We > 100$) for Aerogel. Asai et al.'s model underestimates β_{max} on Aerogel when We is above 100.

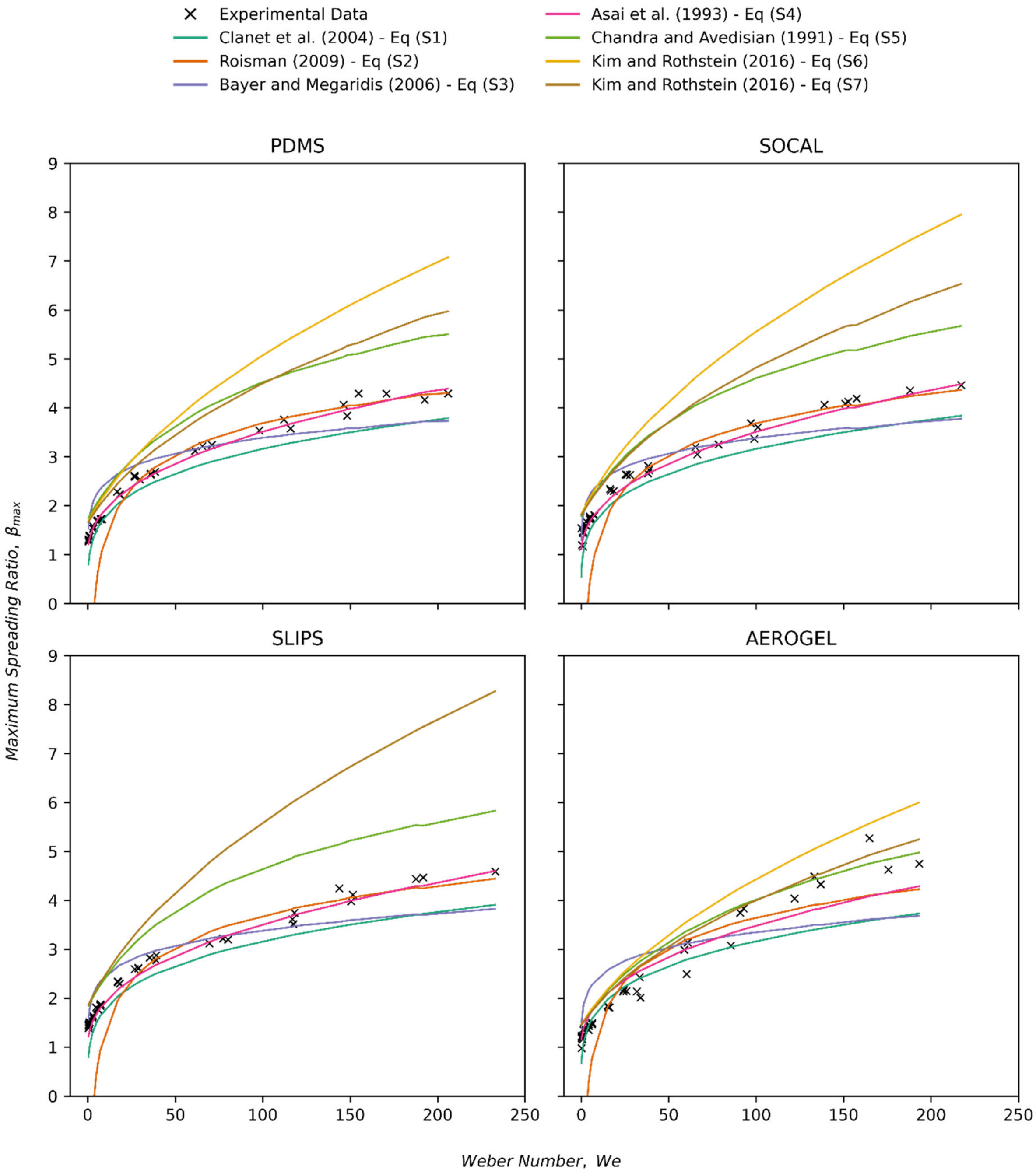

Figure S2. Graphs showing comparisons between the predictions of β_{max} made by the models described in equations S1-S7, and the experimental results for β_{max} collected across the four different surfaces tested in this study.

References

[1] C. Clanet, C. BÉGuin, D. Richard, D. QuÉRÉ, Maximal deformation of an impacting drop, Journal of Fluid Mechanics 517 (2004) 199-208.
[2] I.V. Roisman, Inertia dominated drop collisions. II. An analytical solution of the Navier–Stokes equations for a spreading viscous film, Physics of Fluids 21(5) (2009) 052104.
[3] I.S. Bayer, C.M. Megaridis, Contact angle dynamics in droplets impacting on flat surfaces with different wetting characteristics, Journal of Fluid Mechanics 558 (2006) 415-449.
[4] A. Asai, M. Shioya, S. Hirasawa, T. Okazaki, Impact of an ink drop on paper, Journal of imaging science and technology 37 (1993) 205-205.
[5] S. Chandra, C.T. Avedisian, On the collision of a droplet with a solid surface, Proceedings of the Royal Society of London. Series A: Mathematical and Physical Sciences 432(1884) (1991) 13-41.
[6] J.H. Kim, J.P. Rothstein, Droplet Impact Dynamics on Lubricant-Infused Superhydrophobic Surfaces: The Role of Viscosity Ratio, Langmuir 32(40) (2016) 10166-10176.
